# Genome-wide profiling of transcription factor activity in primary liver cancer using single-cell ATAC sequencing

**DOI:** 10.1016/j.celrep.2023.113446

**Published:** 2023-11-18

**Authors:** Amanda J. Craig, Maruhen A. Datsch Silveira, Lichun Ma, Mahler Revsine, Limin Wang, Sophia Heinrich, Zachary Rae, Allison Ruchinskas, Kimia Dadkhah, Whitney Do, Shay Behrens, Farid R. Mehrabadi, Dana A. Dominguez, Marshonna Forgues, Anuradha Budhu, Jittiporn Chaisaingmongkol, Jonathan M. Hernandez, Jeremy L. Davis, Bao Tran, Jens U. Marquardt, Mathuros Ruchirawat, Michael Kelly, Tim F. Greten, Xin W. Wang

**Affiliations:** 1 Laboratory of Human Carcinogenesis, Center for Cancer Research, National Cancer Institute, Bethesda, MD 20892, USA; 2 Cancer Data Science Laboratory, Center for Cancer Research, National Cancer Institute, Bethesda, MD 20892, USA; 3 Liver Cancer Program, Center for Cancer Research, National Cancer Institute, Bethesda, MD 20892, USA; 4 Department of Gastroenterology, Hepatology, Infectious Diseases and Endocrinology, Hanover Medical School, 30159 Hanover, Germany; 5 Frederick National Laboratory for Cancer Research, Leidos Biomedical Research, Inc., Frederick, MD 20701, USA; 6 Department of Surgical Oncology, City of Hope National Medical Center, Duarte, CA 91010, USA; 7 Laboratory of Chemical Carcinogenesis, Chulabhorn Research Institute, Bangkok 10210, Thailand; 8 Center of Excellence on Environmental Health and Toxicology, Office of Higher Education Commission, Ministry of Higher Education, Science, Research and Innovation, Bangkok 10400, Thailand; 9 Surgical Oncology Program, Center for Cancer Research, National Cancer Institute, Bethesda, MD 20892, USA; 10 Department of Medicine I, University of Lübeck, 23552 Lübeck, Germany; 11 Thoracic and GI Malignancies Branch, Center for Cancer Research, National Cancer Institute, Bethesda, MD 20892, USA; 12 Lead contact

## Abstract

Primary liver cancer (PLC) consists of two main histological subtypes; hepatocellular carcinoma (HCC) and intrahepatic cholangiocarcinoma (iCCA). The role of transcription factors (TFs) in malignant hepatobiliary lineage commitment between HCC and iCCA remains underexplored. Here, we present genome-wide profiling of transcription regulatory elements of 16 PLC patients using single-cell assay for transposase accessible chromatin sequencing. Single-cell open chromatin profiles reflect the compositional diversity of liver cancer, identifying both malignant and microenvironment component cells. TF motif enrichment levels of 31 TFs strongly discriminate HCC from iCCA tumors. These TFs are members of the nuclear/retinoid receptor, POU, or ETS motif families. POU factors are associated with prognostic features in iCCA. Overall, nuclear receptors, ETS and POU TF motif families delineate transcription regulation between HCC and iCCA tumors, which may be relevant to development and selection of PLC subtype-specific therapeutics.

## In brief

Craig et al. profile transcription factor enrichment in 16 patients with liver cancer using single-cell assay for transposase accessible chromatin sequencing (scATAC-seq). The authors find a 31 transcription factor motif enrichment signature that distinguishes HCC from iCCA tumors. Enrichment of the POU motif family is associated with poor prognosis in iCCA.

## Graphical abstract

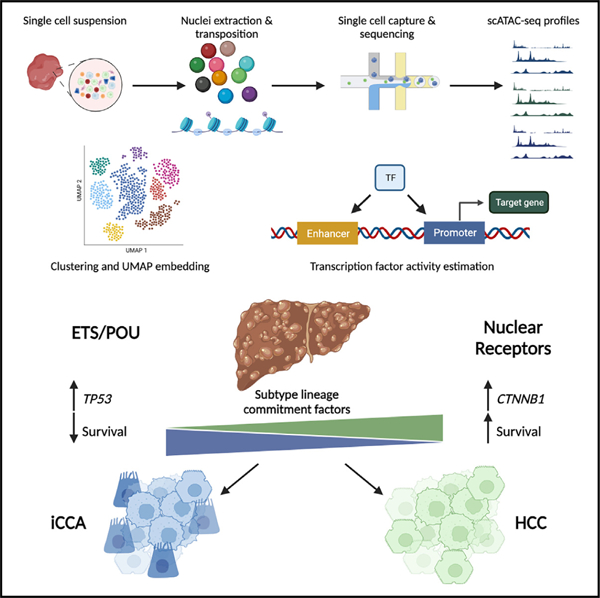

## INTRODUCTION

Hepatocellular carcinoma (HCC) and cholangiocarcinoma (CCA) are the two main forms of primary liver cancer (PLC). HCC accounts for over 90% of PLC cases and typically arises in the background of chronic liver disease induced by viral hepatitis, alcohol consumption, fatty liver disease, and other metabolic syndromes.^1^ Intrahepatic cholangiocarcinoma (iCCA) also develops within the liver and is associated with dismal outcomes and a 5-year survival of less than 10%.^2^ iCCA shares several risk factors with HCC, including chronic liver disease induced by viral hepatitis.^3^ While hepatocytes are believed to be the source of a majority of HCCs and cholangiocytes for iCCAs, there are many studies that show evidence for trans-differentiation of hepatocytes to biliary-like cells to form iCCA and hepatic progenitor cells as the origin for both tumor types.^4,5^ Recently, it was shown that oncogenic MYC acts as a switch between HCC and iCCA development.^6^ High levels of MYC in *Kras*^G12D^-transformed hepatocytes led to the development of HCC, while normal levels of MYC led to iCCA development. MYC regulates this switch by interacting with the transcription factors (TFs) ETS1 and FOXA1/2 to promote transcription of hepatocyte lineage determination genes. On the other hand, MYC represses cholangiocyctic lineage commitment factor transcription through interfering with ETS1 binding to transcriptional machinery. In light of these findings, we aim to comprehensively profile the global landscape of TF motif enrichment and their relationship to hepatobiliary lineage commitment factors in PLC.

TFs are important regulators of lineage determination.^7^ Mechanistically, TFs bind to specific DNA sequences, or motifs, and influence transcription through protein-protein interactions and by recruitment of the transcriptional apparatus to specific genes.^7^ Furthermore, the concept of transcriptional addiction in cancer suggests that cancer cells are highly sensitive to the loss of function of specific TFs, illustrating the complexity of TFs governing specific cell fate.^8^ Progenitor liver cells are bipotent and can develop into either hepatocytes or biliary epithelial cells.^9^ In normal physiology, key TFs control the transcription of lineage determination pathways,^10^ imposing transcriptional programs to control cell fate. The TFs HNF4/HNF1α and HNF6/HNF1β are critical for cell-type-specific transcriptional programming of hepatocytes and cholangiocytes, respectively.^9^ The intricacies of transcription regulation in cancer are an expanding area of research. New technologies such as the assay for transposase accessible chromatin sequencing (ATAC-seq) allow for genome-wide profiling of transcription regulatory elements.^11^ Importantly, ATAC-seq is well suited for single-cell platforms, allowing us to separate malignant from non-malignant components of tumors and analyze them separately.^12^ With this technology, we can now estimate the motif enrichment of all TFs on a single-cell basis in PLC.

Considering the dynamic role of TFs regulating cell determination transcriptional programs, we asked which TF motifs are enriched in PLC and how these TFs impact tumor biology. Here, we present findings of single-cell ATAC sequencing (scATAC-seq) of 16 PLC patients. We estimated TF motif enrichment in malignant cells from both HCC and iCCA tumors and discovered a signature of 31 TFs that accurately distinguish HCC from iCCA tumors. These TFs belong to the nuclear/retinoid receptor (NR), ETS, and POU motif families. NR and ETS factors are important for maintaining transcription of hepatocytic and cholangiocytic lineage determination genes, respectively.^9^ Interestingly, enrichment of POU motifs in iCCA tumors was associated with poor prognosis in iCCA patients, representing a potential therapeutic target.

## RESULTS

### Single-cell whole-genome accessibility profiles determined by scATAC-seq reflect cell composition diversity of PLC

**Figure 1.**
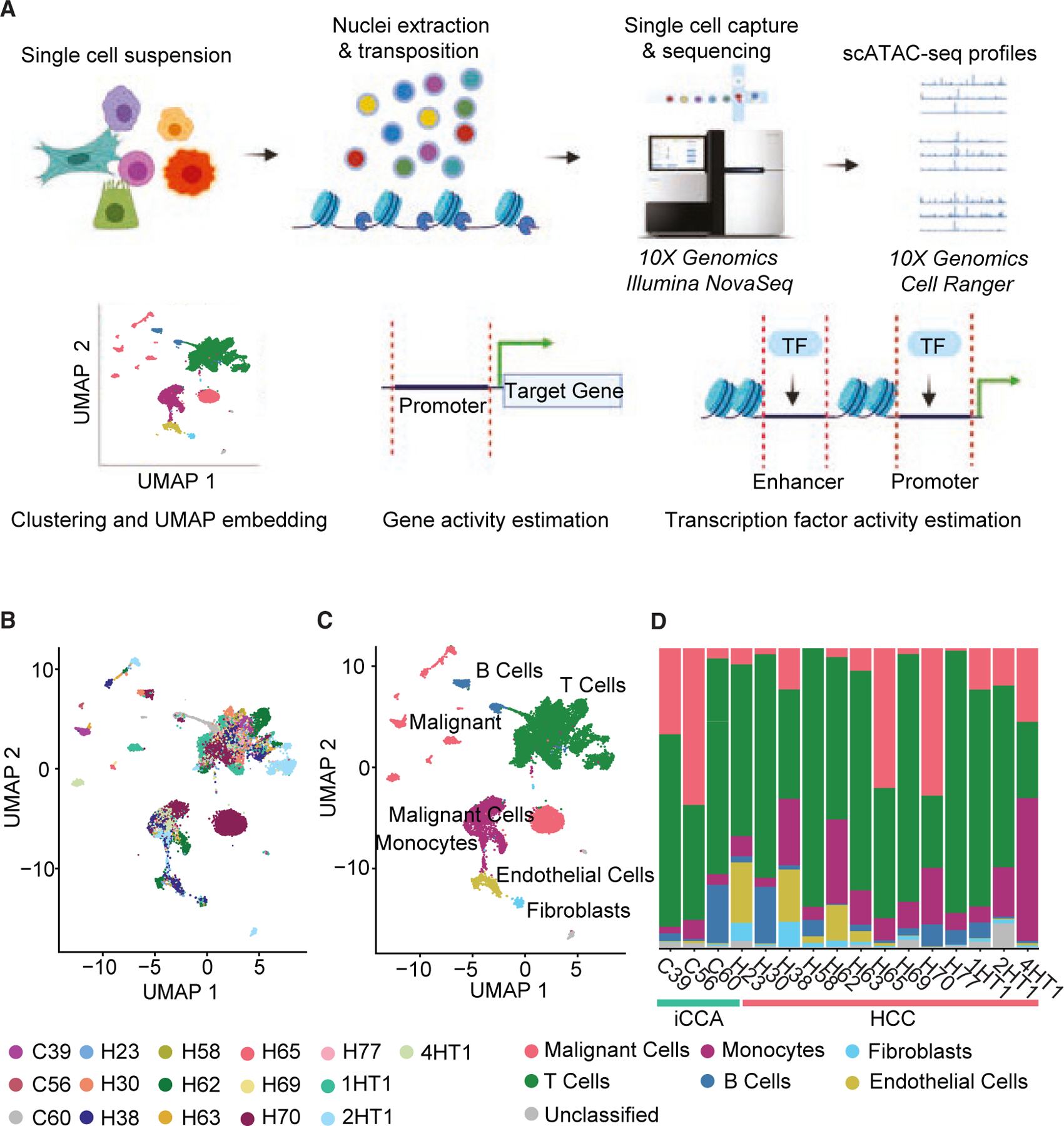
Single-cell chromatin accessibility of 18,631 cells from 16 primary liver tumors (A) Schematic presentation of experimental and analytical workflow. (B) UMAP visualization of all 18,631 cells. Patient IDs start with H and C to denote the clinical diagnosis of HCC and iCCA, respectively. Each color represents an individual patient. (C) UMAP visualization of all 18,631 cells. Each color represents a cell type. (D) Relative abundance of cell type in each patient. See also Figures S1‒S4.

To comprehensively assess chromatin accessibility and transcription regulation in PLC, we performed scATAC-seq on 16 patients, consisting of 13 HCC and 3 iCCA. Mean age of patients at time of sample collection was 67 years with a slight male predominance (63%). Patients ranged from early- to late-stage disease and etiologies included HBV, HCV, and metabolic disease (Table 1). Patient samples were collected at surgical resection or prior to immunotherapy treatment with percutaneous biopsy. The experimental approach for performing scATAC-seq is outlined in Figure 1A (see STAR Methods). We obtained chromatin accessibility profiles from 18,631 cells that passed quality control metrics (Figures S1A–S1C; Table S1, see STAR Methods). To validate the hepatic origin of the cells, we interrogated the promoter region of a housekeeping gene (*GAPDH*) as a positive control, and a brain-specific gene (*SFTPA2*) as a non-liver-related negative control and observed a strong peak in the gene body and upstream region of *GAPDH* across all compiled cells and a noticeable absence of any peaks in these regions of *SFTPA2* (Figure S1D). Peaks were mainly located in distal intergenic or other intronic regions, while 21.77% of peaks were in promoter regions (<3 kb upstream of transcription start sites, Figure S1E). For normalization and dimension reduction, we performed latent semantic indexing (LSI), running singular value decomposition (SVD) using the top 5% most present peaks for feature input (see STAR Methods). For visualization, we used the first 10 LSI components to perform UMAP dimension reduction. Graph-based clustering identified 33 clusters, some of which were dominated by individual patients while others were composed of cells from multiple patients (Figures 1B and S1F).

To classify each cluster by cell type, we utilized our previously published scRNA-seq data performed on the same cohort of patients. Using our matched annotated scRNA-seq data as the reference dataset, we transferred cell type labels onto our scATAC-seq data using the Signac R package.^13^ This required first the generation of gene enrichment scores for each gene in each cell of the scATAC-seq dataset to be used as surrogate gene expression values. Gene enrichment scores are calculated by quantifying open peaks within the gene body and 2 kb upstream regions of each gene followed by normalization. Now having equivalent assays for comparison purposes, anchorsets between the reference scRNA-seq dataset and the query scATAC-seq gene enrichment estimation dataset were calculated. Anchorsets represent matching cell pairs with similar biological states. Cell type annotations of the scRNA-seq are then transferred to the scATAC-seq component cells based on nearest neighbors to these pairwise correspondences. A total of 98.75% of cells was predicted to have a highly accurate predicted label (prediction accuracy score >0.50, Figure S2A; Table S2). With further refinement of cell labels by cluster, we identified malignant cells (n = 4,184), T cells (n = 9,187), monocytes (n = 3,177), B cells (n = 930), endothelial cells (n = 618), and fibroblasts (n = 281, Figure 1C). T cell, monocyte, and malignant (epithelial) predictions were also confirmed by the enrichment of cell-type-specific gene signatures within each cell population cluster, respectively (Figure S2B). Furthermore, peak enrichment at the promoter region of *ALB*, a liver marker gene, was most prominent in the predicted malignant cells (Figure S2C). To ensure predicted malignant cells were indeed malignant and not just epithelial cells, we estimated copy number variations for each cell as previously described and observed a significant increase in our predicted malignant cells compared with other cell type populations (Figure S3).^14^ As previously described, malignant cells cluster separately by patient, whereas microenvironment component cell clusters were intermixed, consisting of cells from multiple patients.^15^ Comparing the distribution of cell type composition across patients, we observed that a majority of cells sampled from each patient were T cells, with abundant variation in the detection of malignant cells across patients (Figure 1D; Table S2).

Next, we compared our predicted populations and identified cell-type-specific peaks (Figure S4A; Table S3). The nearest genes to these identified peaks were assessed by gene ontology enrichment. Top gene paths identified included a majority of the liver cell type signatures described by Aizarani et al., including the enrichment of liver microvascular endothelial cell signature in the endothelial cell population and enrichment of the liver Kupffer cell signature in the monocyte population (Figure S4B).^16^ Finally, we estimated TF motif enrichment for each cell based on the fraction of peaks that contained TF binding motifs using the ChromVAR R package.^17^ Peaks of each cell were first annotated for the presence of TF motif binding regions. TF motif enrichment scores were then determined by calculating deviations in actual chromatin accessibility at TF motif binding regions from the expected accessibility based on the average across all cells. Again, we were able to identify uniquely enriched TF motifs for different cell types, several of which have been previously described as playing important roles in those cell types’ differentiation, such as HNF4G in malignant cells and IRF4 in B cells (Figures S4C and S4D; Table S4). Interestingly, we found very few TF motifs solely enriched in T cells and fibroblasts. These results imply that any TF motif enriched in T cells or fibroblasts was also enriched in a different cell type. In contrast to this observation in TF motif enrichment, there were in fact many open chromatin peaks uniquely enriched in T cells and fibroblasts that did not include TF binding motifs (Figures S4A and S4D). This suggests that uniquely enriched peaks may be a better way to identify T cells and fibroblasts from scATAC-seq profiles.

**Figure 2.**
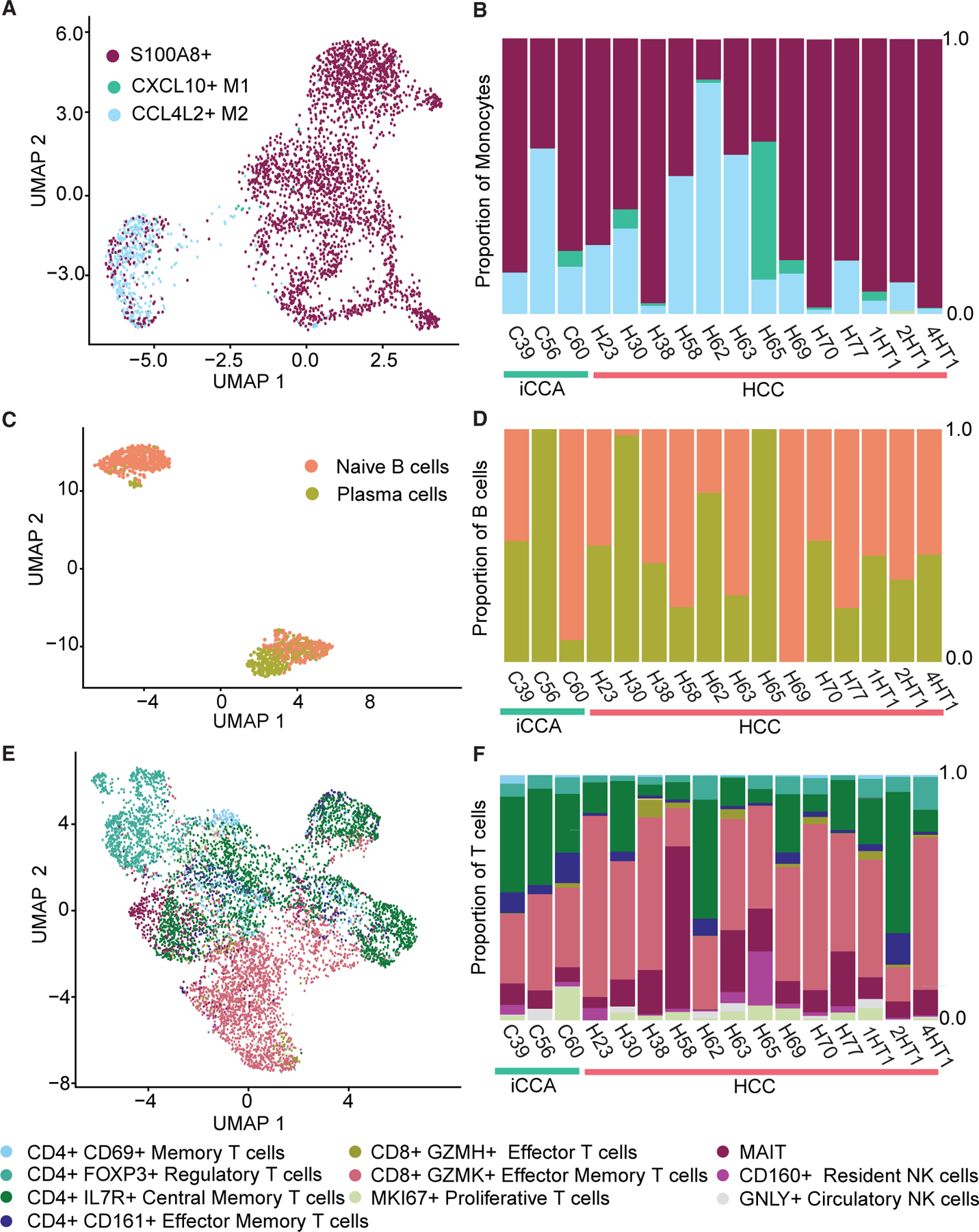
Microenvironment analysis in scATAC-seq (A) UMAP visualization of monocytes. Each color represents a cell subtype. (B) Relative abundance of monocyte subtypes in each patient. (C) UMAP visualization of B cells. (D) Relative abundance of B cell subtypes in each patient. (E) UMAP visualization of T cells. (F) Relative abundance of T cell subtypes in each patient. See also Figures S5‒S7.

Along with others, we have previously demonstrated that the interaction between the tumor microenvironment and malignant cells plays a profound role on the phenotype of tumors.^15,18^ This prompted us to further characterize the microenvironment component cells. We again used our matched scRNA-seq data to transfer annotation data, this time identifying cell subtypes for each component of the microenvironment. Less than 100 fibroblast or endothelial cells were captured, and the subtype prediction scores were too low to be deemed reliable. This finding was unexpected, as iCCA tumors often have a substantial stromal compartment and may be due to technical limitations from tumor dissociation.^19^ In the case of monocytes, very few were captured from patients with iCCA and thus we were unable to do a subtype level comparison of these cells between tumor subtypes. Monocyte detection trended higher in HCC, although several patients with HCC had low levels of monocytes similar to the patients with iCCA. This suggests that monocyte infiltration may be more patient rather than tumor subtype driven, or a technical problem may be impeding our detection of monocytes. Nevertheless, monocyte subtypes were detected in several patients with HCC, including S100A8^+^, CXCL10^+^ M1, and CCL4L2^+^ M2 populations (Figures 2A, 2B, and S5A). We further focused on B and T cells, for which we captured a substantial number of cells from both patients with HCC and iCCA. We observed high inter-patient heterogeneity of T and B cell subtype composition, with no significant enrichment of any identified subtype in either HCC or iCCA tumor types. B cells were divided into two populations with markers of either naive or plasma cells (Figures 2C and S5B). All tumors contain a mixture of both naive and plasma B cells except for patient H69, for whom we only identified naive B cells, and patients C56 and H65, for whom we only identified plasma B cells (Figure 2D). Within the T cells, both CD4^+^ and CD8^+^ populations were detected. A majority of the CD4^+^ population were IL-7R^+^ central memory T cells (Figures 2E and S5C). The second most prominent CD4^+^ population was FOXP3^+^ regulatory T cells, while a much smaller percentage was either CD69^+^ memory T cells or CD161^+^ effector memory T cells. The CD8^+^ T cell population was predominantly GZMK^+^ effector memory T cells. In addition, we detected small numbers of MKI67^+^ proliferative T cells, CD160^+^ resident NK cells, and GNLY^+^ circulatory NK cells (Figure 2F). Finally, a large population of MAIT cells were detected in one patient (patient H58). Overall, open chromatin profiling captured the full breadth of the PLC microenvironment, which broadly overlapped between HCC and iCCA tumors. The lack of any significant B or T cell subtype enrichment by PLC subtype was confirmed in an additional 24 patients (33 in total including patients from the scATAC-seq cohort) using scRNA-seq data for cell type annotation (Figure S6). This may reflect the high variation in immune composition of both HCC and iCCA tumors and the need for further classification of both tumor types by immune cell status. We also investigated tumor stage as a variable that may be associated with immune infiltrate. When comparing the cell type proportions (percent of total detected per patient) between early- (N = 6) and late-stage tumors (N = 9), we do see significant differences or trends in cell type composition (Figure S7). The proportion of T cells was significantly higher in late-stage tumors (early-stage median = 0.45, late-stage median = 0.721, p = 0.05), while the proportion of monocytes detected was significantly higher in early-stage tumors (early-stage median = 0.21, late-stage median = 0.06, p = 0.0076). This shows that there are many influences on immune infiltrate aside from PLC subtype and these should also be considered when making comparisons between HCC and iCCA. In summary, these findings suggest that scATAC-seq is sensitive enough to annotate the cellular composition of PLC and identified no significant difference in B or T cell subtype composition between HCC or iCCA, although higher-grade tumors of both PLC subtypes trended toward having higher T cell infiltration and lower monocyte infiltration.

### Unique TF motif enrichment profiles distinguish HCC from iCCA malignant cells

**Figure 3.**
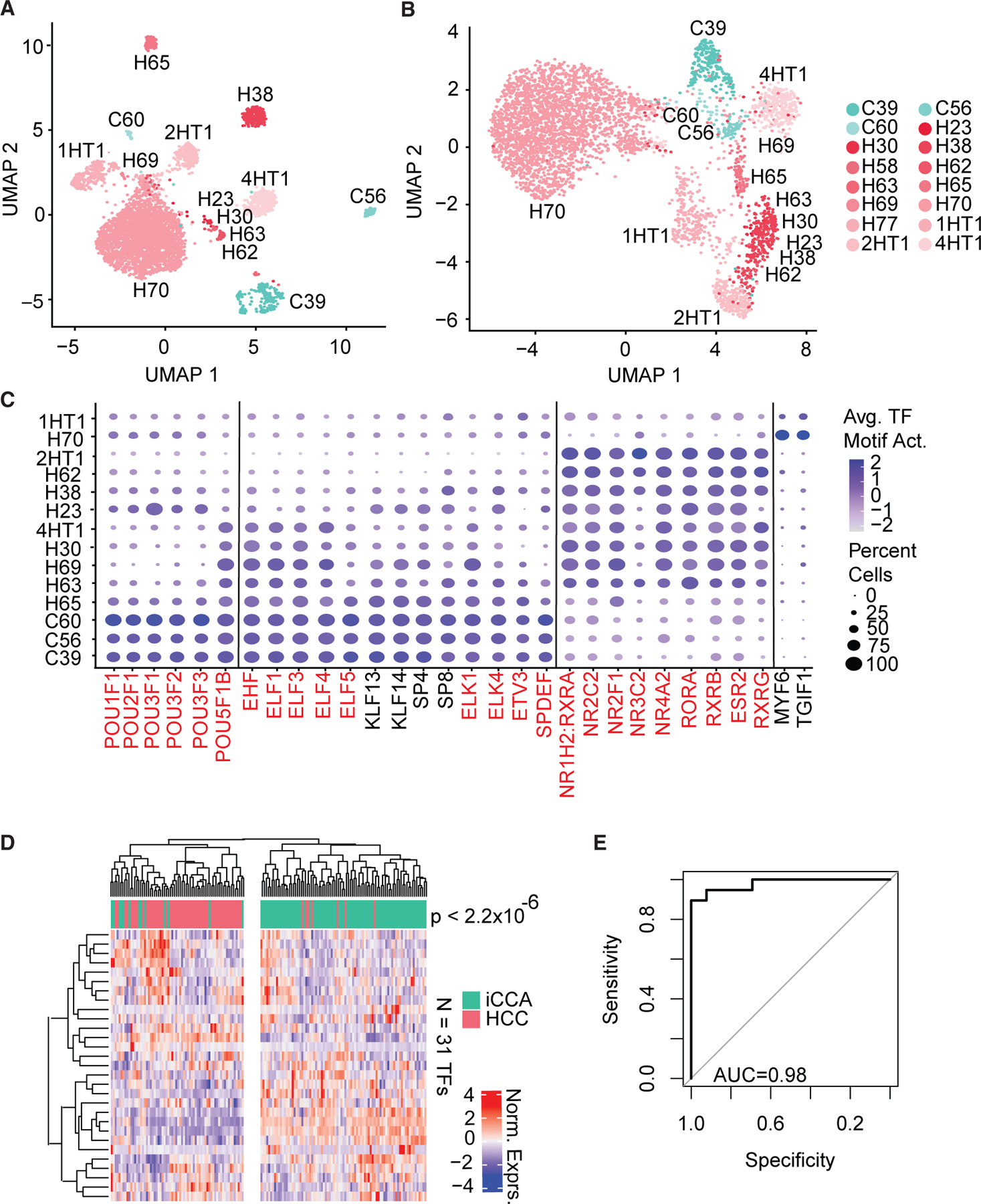
TF motif enrichment differences between HCC and iCCA tumors (A) UMAP visualization of all malignant cells using open peaks as features for dimensionality reduction and clustering. (B) UMAP visualization of all malignant cells using TF motif enrichment as features for dimensionality reduction and clustering. (C) Dot plot showing TF motif enrichment markers for each tumor subtype. NR, POU, or ETS family members are highlighted in red. (D) Hierarchical clustering of TIGER cohort gene expression of 31 TFs. Samples are labeled with tumor subtype. (E) ROC curve of TF motif signature for prediction of tumor subtype. See also Figure S8.

While HCC and iCCA tumors are distinct malignancies, both develop within the liver and share several risk factors.^20^ This along with varying reports regarding the cell of origin for PLCs led us to investigate the differences in the oncogenic transcription program regulation between HCC and iCCA tumors.^4^ To assess this, we repeated dimensionality reduction and clustering on malignant cells alone after running SVD using the top 5% of present peaks and the first 10 LSI as input for UMAP. We observed a clear separation of malignant cells by patients with no discernable separation between HCC- or iCCA-derived cells (Figure 3A). Strikingly, when we instead ran clustering using the top variable (n = 127) TF motif enrichment across all malignant cells, we observed a separation between HCC- and iCCA-derived cells, mainly along the UMAP 2 axis (Figure 3B). Top variable gene enrichment was not similarly driven by differences between HCC and iCCA malignant cells (Figure S8A). This suggests that peaks containing TF binding sites are most influential in driving the variation between HCC and iCCA malignant cells.

This prompted us to identify the most prominent TF binding sites that are accessible between HCC and iCCA malignant cells. We compared TF motif enrichment between malignant HCC and iCCA cells and discovered 31 significant differentially enriched TF motifs (Log2FC > 0.2, FDR < 0.1, Figures 3C and S8B; Table S5). iCCA was enriched for POU and ETS factors, while HCC was dominated by nuclear receptors (NRs). When malignant cells are hierarchically clustered using the 31 TF motif signature, cells dominantly clustered by tumor type (Figure S8C). To see if these findings translate past our scATAC-seq cohort of patients, we utilized the TIGER-LC cohort, for which we have previously published transcriptome profiles for 153 patients with PLC (HCC = 62, iCCA = 91). Hierarchical clustering using the expression of the 31 TF motif signature clusters patients into 2 groups, significantly dominated by HCC and iCCA, respectively (p < 2.2 × 10^−6^, Figure 3D). When assessing the 31 TF motif signature in both tumoral and matching adjacent liver tissue, clustering based on expression patterns separated tumoral from adjacent liver tissue (Figure S8D). Separation of HCC from iCCA tumor samples was still observed, but notably no difference in expression of matching adjacent liver samples was seen and appears independent of whether the patient was diagnosed with HCC or iCCA. Since TFs regulate several downstream genes, we also looked at the expression of 13 tumor-type-specific TFs for which previously published target gene lists were available. Hierarchical clustering of the average of each TF’s target genes again separated patients into iCCA- and HCC-dominated clusters (p < 2.2 × 10^−6^, Figure S8E). An AUC of 0.947 was observed when assessing the 31 TF motif signature as a classifier of HCC or iCCA tumor subtypes by plotting an ROC curve (Figures 3E and S8F). This confirms that the TF motif signature distinguishes HCC from iCCA tumors.

**Figure 4.**
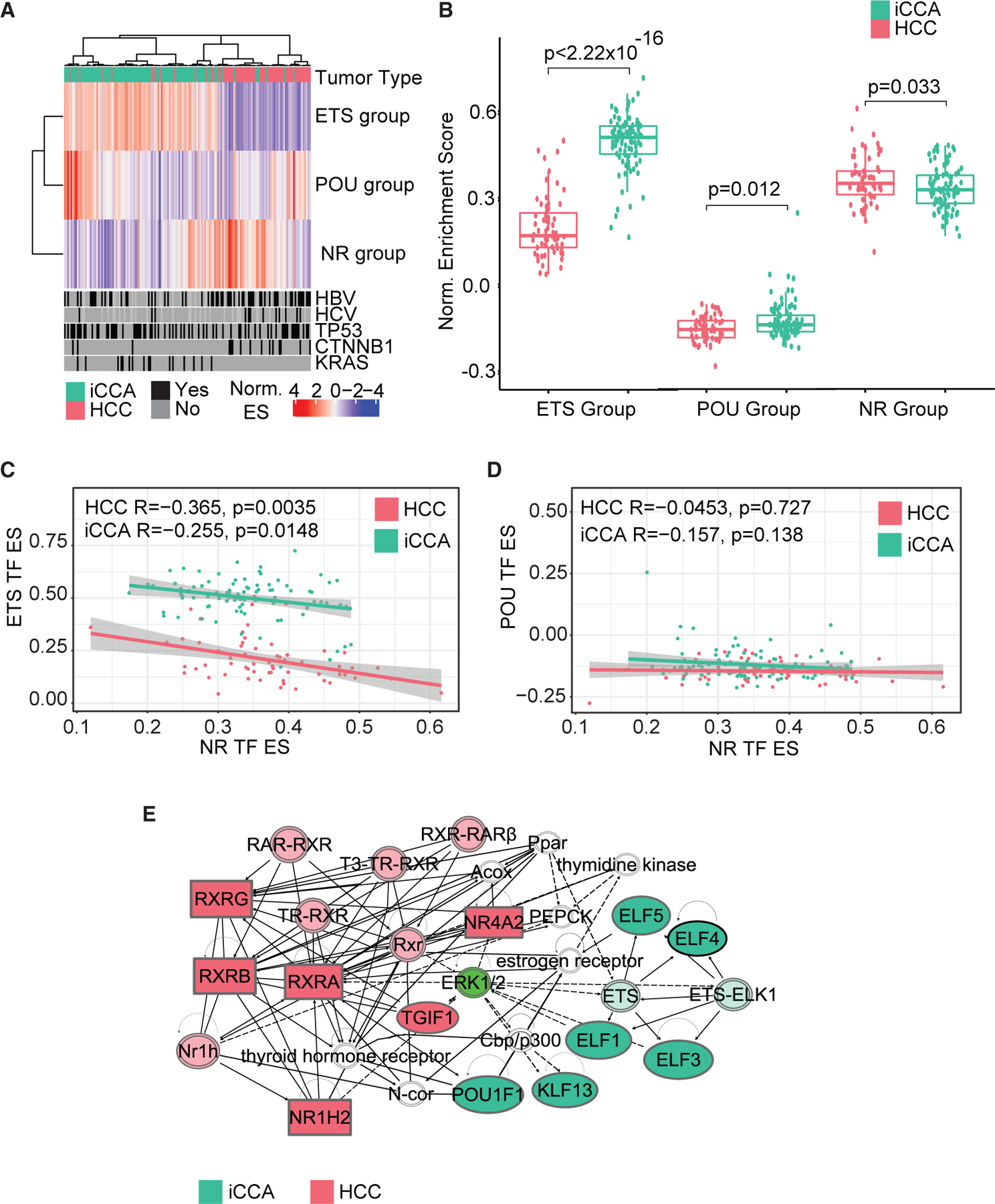
NR, ETS, and POU factors expression in primary liver cancer (A) Hierarchical clustering of TIGER-LC cohort ETS, NR, and POU ES. (B) Boxplots of ETS, POU, and NR ES scores of TIGER-LC cohort separated by tumor subtype. For each boxplot, the center line represents the median. Upper and lower limits of each box represent the 75th and 25th percentiles, respectively. (C) Correlation plot between NR ES and ETS ES in TIGER-LC cohort. (D) Correlation plot between NR ES and POU ES inTIGER-LC cohort. (E) Ingenuity pathway analysis generated a network of 31 TFs. Statistical significance of boxplots was calculated using the Wilcoxon test. Statistical significance of correlation plots was calculated using the Spearman’s rank correlation test. See also Figure S8.

We next investigated the variation in expression of the TF motif signature in the same TIGER-LC cohort (HCC = 62, iCCA = 91). As the iCCA-enriched TFs dominantly fell into either the POU or ETS motif families, we separated these genes into two groups to compare the contribution of each family to the discrimination of HCC from iCCA tumors. For bulk RNA-seq samples, TF enrichment scores were generated using ssGSEA, which calculates normalized difference in empirical cumulative distribution functions of gene expression ranking of genes within or without the gene set of interest.^21^ While the expression of the POU group (N = 6) was significantly higher in iCCA than HCC (p = 0.012), within iCCA tumors expression of the POU group was highly variable (Figures 4A and 4B; Table S6). In comparison, the ETS (N = 13) and NR (N = 9) groups were homogeneously expressed in iCCA and HCC tumors, respectively (p = <2.22 × 10^−6^ and 0.033, Figure 4B; Table S6). Next, we compared the average expression of the POU and ETS groups to the NR motif family group identified as enriched in HCC across all tumors of the TIGER-LC cohort. While no correlation of expression was found between the expression of the POU and NR groups, the expression of the NR and ETS groups was significantly inversely correlated in both HCC and iCCA tumor populations (HCC R = −0.365, p = 0.0035, iCCA R = −0.255, p = 0.0148, Figures 4C and 4D; Table S6). Furthermore, Ingenuity Pathway Analysis on the TF motif signature generated a network connecting NR factors to ETS factors through an ERK1/2 intermediate (Figure 4E; Table S5). Overall, our findings suggest that NR and ETS factors strongly discriminate HCC from iCCA tumors and may interact to create a gradient of PLC subtype identity.

### Heterogeneity of TF motif enrichment in PLC malignant cells

**Figure 5.**
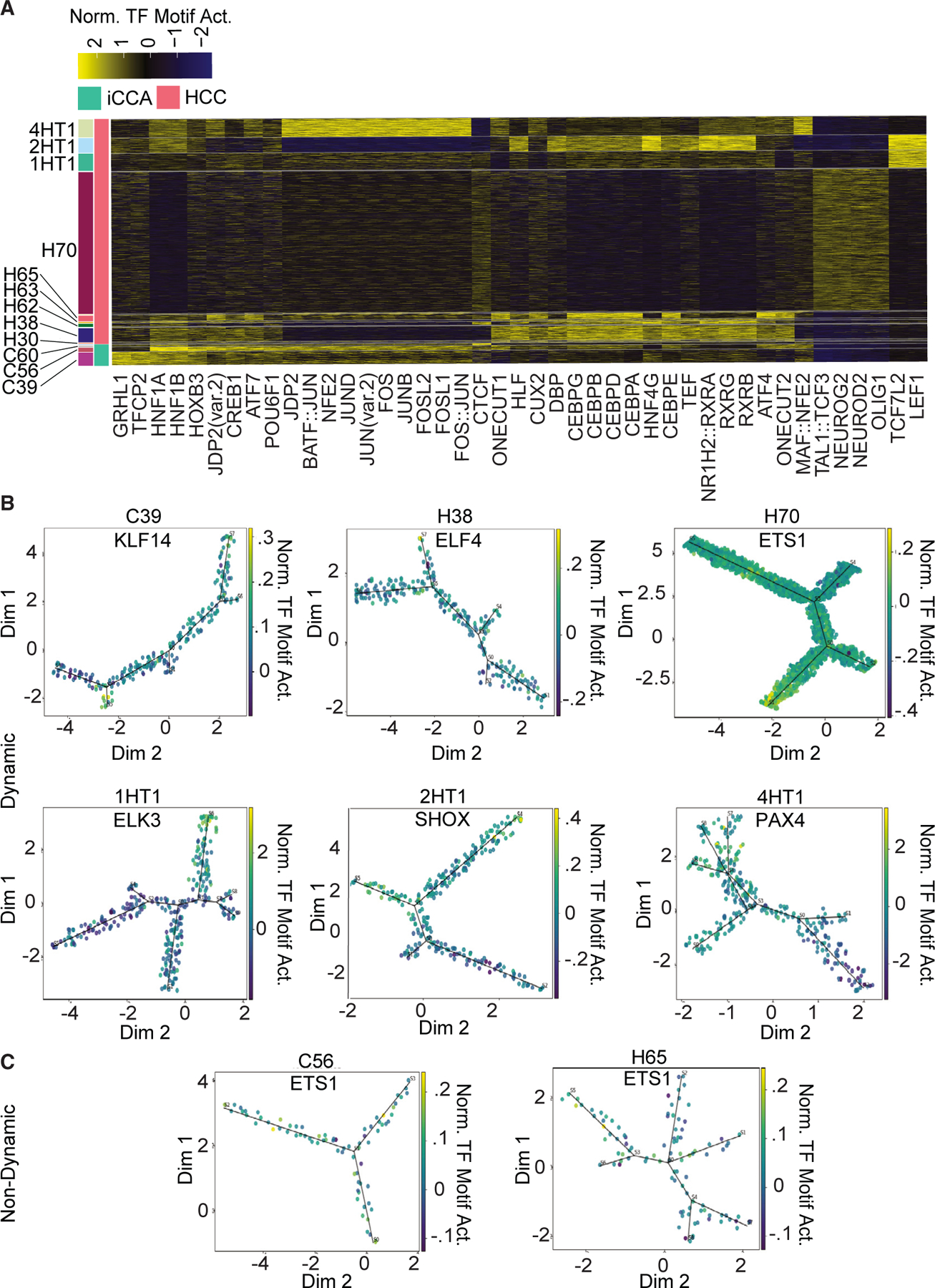
Inter- and Intra-patient heterogeneity of TF motif enrichment (A) Heatmap of TF motif enrichment markers for each patient. (B) Trajectory trees of malignant cells from patients with dynamic changes in TF motif enrichment. Cells are labeled by TF motif enrichment. (C) Trajectory trees of malignant cells from patients with no dynamic changes in TF motif enrichment. Cells are labeled by TF motif enrichment.

PLC is well known as a genetically heterogeneous disease due to a high level of interpatient heterogeneity of driver mutations.^22^ We next decided to test if this interpatient heterogeneity also existed with TF motif enrichment. We identified the most enriched TF motifs for each patient and compared them across the entire cohort (Figure 5A; Table S7). Patient H70 exhibited the most unique TF motif enrichment profile, with enrichment of the neuronal TF motifs NEUROG2, NEUROD2, and OLIG1 and a notable absence of TF motif enrichment that was observed in other patients. HNF1A and HNF1B enrichment was present in all patients, except for patient H70. FOS/JUN-related TF motifs were highly enriched specifically in patients with iCCA and a single patient with HCC, 4HT1. Conversely, CEBP-related TF motifs were highly enriched in a majority of patients with HCC. TCF7L2 and LEF1 TF motifs were uniquely enriched in patients 1HT1 and 2HT1.

Our previous work has shown that a subset of PLCs have high levels of gene expression intra-tumor heterogeneity.^15^ Consistently, high levels of intra-patient heterogeneity of TF motif enrichment revealed by scATAC-seq was also observed. To identify dynamically enriched TFs, we performed trajectory analysis for each patient with an adequate number of malignant cells and identified TF motifs that show gradient changes of enrichment in conjunction with pseudotime changes. Two patients (C56 and H65) show no dynamic changes in TF motif enrichment across all malignant cells, while the other six patients had several TF motifs that had gradient changes in enrichment along a fitted tree axis (p < 0.05, Figures 5B and 5C; Table S8). Interestingly, several of the top dynamically changing TF motifs in patients with HCC were members of the ETS family, which was also identified as highly and homogeneously enriched in patients with iCCA. Notably, branching patterns between the fitted trees of the eight patients analyzed varied in the number of branch points, ranging from two to four, suggesting varying degrees of complexity in TF motif enrichment in different tumors. These analyses show that PLC also exhibits substantial inter- and intra-patient heterogeneity at the transcription regulation level.

### NR motif group associates with favorable prognostic features in HCC, while the POU motif group associates with poor prognostic features in iCCA

**Figure 6.**
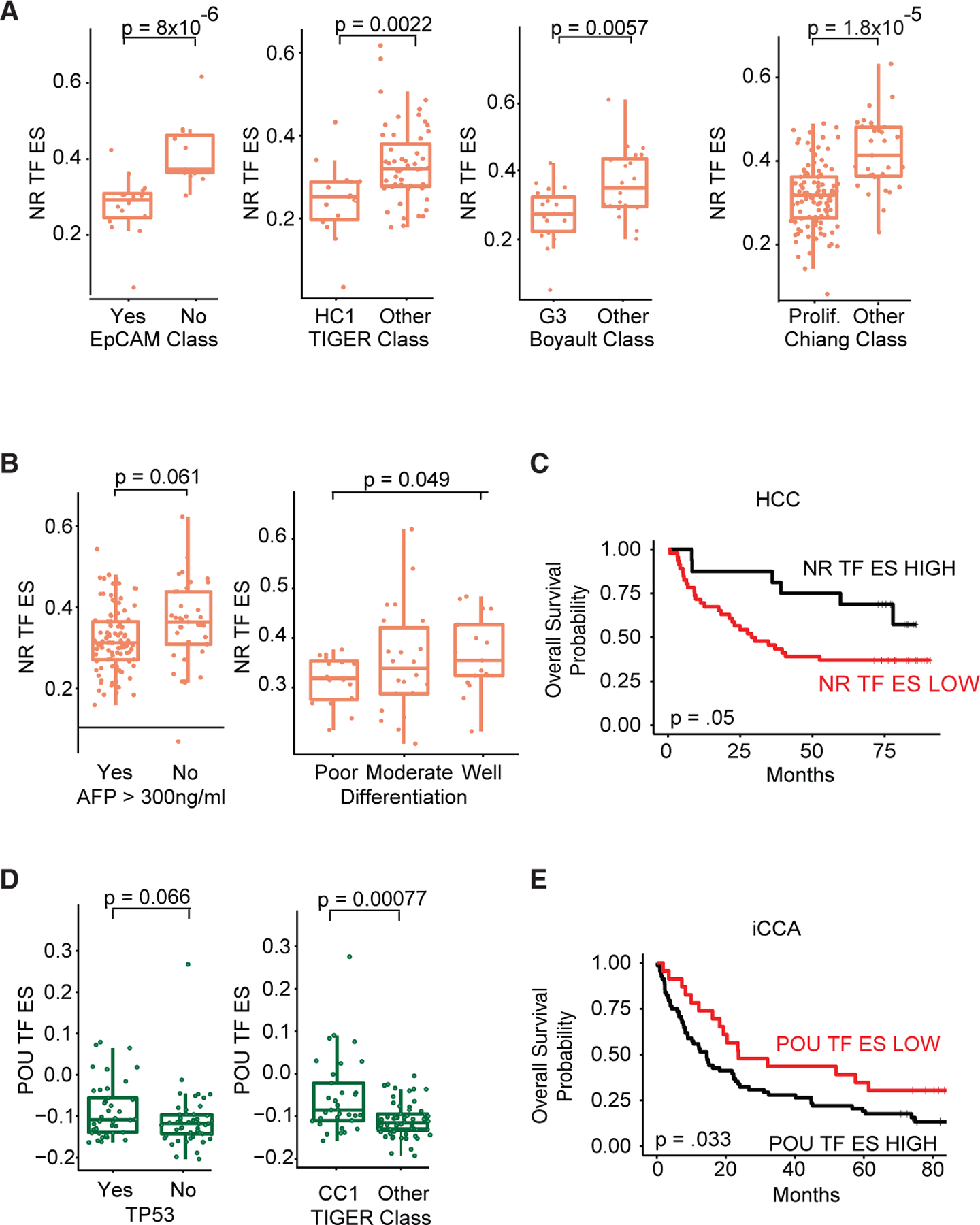
NR and POU factors associate with prognostic factors (A) Boxplots of NR ES scores of TIGER-LC cohort of patients with HCC in different molecular classifications. (B) Boxplots of NR ES scores of the TIGER-LC cohort of patients with HCC with different differentiation statuses and AFP levels. (C) Kaplan-Meier curves showing the percentage of survival between NR ES high and NR ES low TIGER-LC cohort of patients with HCC. (D) Boxplots of POU ES scores of TIGER-LC cohort of patients with iCCA in different molecular classifications. (E) Kaplan-Meier curves showing the percentage of survival between the POU ES high and POU ES low TIGER-LC cohort of patients with iCCA. For each boxplot, the center line represents the median. Upper and lower limits of each box represent the 75th and 25th percentiles, respectively. Statistical significance of boxplots was calculated using the Wilcoxon test or one-way ANOVA test. Statistical significance of Kaplan-Meier curves was calculated using the log-rank test. See also Figure S9.

To further understand the function of the identified tumor-type-specific TF motif families in PLC, we next assessed if there was any association to other molecular or clinical features. NR group enrichment was significantly lower in several previously identified molecular classifications of HCC, including the EpCAM (p = 8 × 10^−6^), HC1 TIGER (p = 0.05), G3 Boyault (p = 0.0057), and Chiang proliferation (p = 2.8 × 10^−8^) classes (Figure 6A; Table S6).^23–26^ Assessing relationships to driver mutations, NR group enrichment was significantly lower in *TP53* mutant tumors (p = 0.0062) but trended higher in *CTNNB1* mutant tumors (p = 0.071, Figures S9A and S9B; Table S6). Clinical features including high AFP levels (>300 ng/mL) and poorly differentiated tumors were also significantly associated with lower NR group enrichment (p = 0.01 and p = 0.049, respectively Figure 6B; Table S6). In agreement with these findings, patients with lower NR group enrichment had a worse overall survival in both the TIGER-LC (p = 0.05) and TCGA-LIHC cohorts (p = 0.032, Figures 6C and S9C; Table S6). We did not find these associations with the NR group in iCCA tumors.

While the ETS group of TFs strongly distinguished iCCA tumors from HCC, they were highly expressed across all iCCA tumors and did not associate with any particular molecular or clinical features. In contrast, the POU group TFs are much more heterogeneously expressed. POU group enrichment trended higher in tumors with *TP53* mutations, were significantly enriched in the CCI TIGER molecular class, and associated with a poorer overall survival (p = 0.066, p = 0.0007, and p = 0.033, respectively Figures 5D and 5E; Table S6). High POU group enrichment was also associated with poor overall survival in the International Cancer Genome Consortium iCCA cohort (p = 0.029, Figure S9D; Table S6). In summary, NR group enrichment was associated with good prognostic features in HCC specifically. In iCCA, while ETS factors are the most distinguishing group of TFs, the more heterogeneously expressed POU factors were associated with poor prognostic features and this association was not seen in HCC.

### Modulation of POU2F1 levels impacts cellular malignant phenotypes in CCA cells

To functionally validate the importance of the POU factor family to cholangiocarcinogenesis, we generated single-cell-derived POU2F1 knockout clones in two independent CCA cell lines. Depletion of POU2F1 was confirmed for HuCCT1 cells (Figure S10A). Migration of POU2F1-depleted cells was drastically reduced compared with control cells (Figure S10B). Also, colony formation was reduced in HuCCT1 cells depleted in POU2F1 compared with control cells (Figure S10C). Similar results were observed in HUH-28 cells (Figures S10D–S10F). Altogether, these results suggest that POU2F1 boosts cellular malignant phenotypes in CCA cells.

## DISCUSSION

Despite significant therapeutic advancements, liver cancer remains a leading cause of cancer-related mortality worldwide.^2^ Both HCC and iCCA arise within the liver and have unique clinical presentations and treatment paradigms. The cornerstone of systemic therapy in HCC treatment relies on immunotherapy, in contrast to iCCA treated with traditional chemotherapy regimens.^27^ Unfortunately, current treatment regimens have limited efficacy, and it is critical to understand the unique development of these malignancies to further guide and understand treatment response. Moreover, rare mixed tumors develop that contain both HCC- and iCCA-like malignant cells.^28^ Recent evidence has suggested that progenitor liver cells can give rise to both HCC and iCCA and even hepatocytes are able to transdifferentiate into cholangiocyte-like cells responsible for iCCA formation.^4^ Knowing what TFs are needed to maintain hepatocytic or cholangiocytic functions in PLC may help us exploit transcriptional networks that drive tumors into a more differentiated state. Here, we show that high levels of heterogeneity in TF motif enrichment exists within and between patients. We demonstrate that the NR and ETS TF motif groups can strongly discriminate HCC from iCCA tumors. In addition, while the NR and POU motif family may be enriched in both patients with HCC and iCCA, they are only associated with prognostic factors in one subtype, suggesting that their role in malignancy is contingent on the presence of hepatocytic or cholangiocytic features.

The tumor microenvironment is known to influence the aggressiveness and differentiation of malignant cells. Important experimental work has shown that the type of cell death that is prominent during liver injury leads to the development of different subtypes of PLC.^29^ Interestingly, no difference in microenvironment composition was observed despite the difference in cell death, suggesting that the influence of the microenvironment may be due to specific cytokine autocrine signaling rather than being dependent on any one cell type. Compared with these results, the only composition differences we observed was a non-significant enrichment of monocytes in HCC compared with iCCA. No differences in B or T cell subtype composition were detected. This is interesting because early immunotherapy trials in PLC suggested that HCC had a better response to immunotherapy than iCCA. But the recent positive TOPAZ-1 trial of durvalumab (anti-PD1 antibody) plus gemcitabine and cisplatin indicates a central role for immunotherapy in the systemic therapy of advanced iCCA.^27^ One reason we may see no difference in microenvironment composition is the high variation of infiltrating immune cells in HCC. Tumor grade may also be a factor, as we also show higher-grade tumors of both PLC subtypes trended toward having higher T cell and lower monocyte infiltration. A more comprehensive analysis of a larger cohort may be needed to subgroup patients as having high or low levels of immune infiltrate before comparisons can be made between subtypes.

PLC is a notoriously heterogeneous disease. In HCC the most recurrent mutations include telomerase promoter, *TP53*, and *CTNNB1* at 60%–90% and 15%–30%, respectively, followed by other genes mutated in only a small subset of patients. Several molecular classifications exist that show high variation of immune infiltrate, signaling pathway activation, and pathologic features.^22^ Here, we add TF enrichment as another heterogeneous aspect of PLC. Each patient had uniquely enriched TFs. H70 showed strong enrichment of NEUROG2 and NEUROD2 that were not seen enriched in any other patient. Some were shared between a subset of patients such as OLIG1/LEF1 in patients 1HT1 and 2HT1, or CEBP motif family that was present in 8/11 patients with HCC. Interestingly, FOS/JUN-related motifs were enriched in both patients with HCC and iCCA, showing that, although distinct pathologic entities, there exists overlap of transcription regulation between a subset of patients. When comparing TF motif enrichment within individual patients, patients could be separated into those who had gradient changes in enrichment, or those who only had constant enrichment across all cells. TF motifs that showed gradient responses included ELK3, ETS1, and DLX6. These gradients may be due to responses from the environment or nutrient constraints causing the tumor to adjust which genes are being transcribed.

Separately, there are TF motif families that strongly distinguish HCC from iCCA malignant cells, suggesting that certain transcriptional programs appear necessary for hepatocyte or cholangiocyte features to be maintained. Clustering analysis of malignant cells by variation in gene expression or TF motif enrichment suggest that TF motif enrichment is better able to distinguish HCC from iCCA. This may be because TFs better reflect cell lineage as specific TFs can direct lineage specification.^6,29^ In contrast, the transcriptome includes many features that are not cell lineage related. Notably, the expression of the NR, ETS, and POU groups were not found to be associated with any driver mutations consistently in both HCC and iCCA, suggesting their expression may be determined more by cellular origin rather than genetic alterations. NRs are highly enriched in HCC tumors and align with favorable prognostic features. These receptors have important roles in regulating functions of normal physiologic hepatocytes, including lipid and xenobiotic metabolism. In fact, there is strong evidence that NRs are tumor suppressive in HCC and expression patterns are repressed compared with normal liver.^30^ Here, we confirm the association of high NR expression with better prognostic markers in HCC but found no association in iCCA. This seems to align with the differentiation status of HCC tumors. Indeed, NR enriched patients are significantly more likely to be well differentiated, probably influencing overall survival. Of note, patient H70 is completely unique and lacks NR motif enrichment almost completely. This may reflect the undifferentiated status of the tumor that is beyond the point of having any remnants of hepatocytic function.

Second, ETS factors overall regulate proliferation and functional features of epithelial cells.^31^ Strikingly, ETS factors are not described in detail in cholangiocytes or iCCA. Upon evaluation of gene markers of cholangiocytes and CCA malignant cells, the ETS factor ELF3 recurrently appears in these lists.^32^ Indeed, ELF3 was already described as a tumor suppressor in CCA and that mutations in ELF3 are usually more associated with perihilar CCA and distal CCA than intrahepatic CCA. In addition, ETS1 was recently shown to be a target of MYC suppression in PLC formation, leading to lower cholangiocytic gene expression and the outgrowth of HCC tumors.^6^ This suggests that ETS factors may play a role in regulating cholangiocytic functions. Interestingly, ETS factors were found as the most dynamic TF motifs in patients with HCC. The presence of dynamically changing epithelial specific ETS factors in HCC may suggest transitions in the epithelial nature of these tumors or addition of iCCA features. The role of ETS factors warrants further investigation to understand their mechanism of action in cholangiocytes, especially due to the difference in recurrent mutations between different locations of CCA.^33^ Finally, the POU motif group was found to be enriched in iCCA tumors. Although POU factors were heterogeneously expressed in both HCC and iCCA tumors, they only associate with prognosis in iCCA. As confirmation, *in vitro* experiments showed CCA cell lines had decreased proliferative and migratory capacity after knockout of POU2F1. The mechanism by which other POU factors contribute to more aggressive tumors with cholangiocytic features deserves further investigation.

In conclusion, we demonstrate distinct TF motif families can distinguish HCC from iCCA and may drive PLC lineage specific transcription patterns using scATAC-seq. We further demonstrate that differential enrichment of TF families is associated with survival in patients with PLC. Overall, scATAC-seq of PLC samples provided valuable insights into the role of TFs in maintaining hepatocytic or cholangiocytic features that appear influential for patient prognosis. Future studies are needed to identify therapeutic vulnerabilities created by aberrant TF motif enrichment in PLC.

### Limitations of the study

We acknowledge several limitations to our study, some of which may hopefully be addressed with improvement to technologies used for single-cell based assays. First, sample acquisition and processing from human patients remains an arduous and time consuming process. Many cells are lost during filtration steps, including rare cell type populations or more fragile cell types such as malignant cells from PLC. This is compounded by small sample sizes obtained by needle biopsies, which account for roughly half the samples included in our study. For these reasons we may be missing rare cell type populations from our analysis. Furthermore, PLC, especially the iCCA subtype, are rare tumor types and thus few patients are enrolled for sample collection. In our study, this led to limitations in generalizing our findings to other patients with iCCA as our results were obtained from only three patients. Given more time, this may be addressed by enrollment and sequencing of more patients. Finally, several assumptions are made when using bioinformatic tools or algorithms for the analysis of scATAC-seq. Indeed, the analysis presented here finds strong associations of TF motif enrichment patterns between PLC subtypes, but this may not necessarily reflect causation. In the analysis of TF motif enrichment, we assume the more TF binding motifs detected in open region peaks, the higher the likelihood of TF binding and downstream influence on target gene expression. Of course, these assumptions do not always hold true. Some factors that may impact TF binding are the number of available TF molecules not being high enough to saturate all available binding sites or a dimerization partner necessary for binding is not present.^7^ Second, RNA levels of TFs are unstable with short half-lives and thus may not correlate with protein levels in every circumstance.^34^ Any hypotheses generated from these analyses must be further functionally validated. Here, we provide evidence in support of the hypothesis that the POU motif family members contribute to cholangiocarcinogenesis using *in vitro* knockout experiments of POU2F1, but further work is needed to fully understand this mechanism of action.

## STAR★METHODS

### RESOURCE AVAILABILITY

#### Lead contact

Further information and requests for resources and reagents should be directed to Dr. Xin Wei Wang (xw3u@nih.gov).

#### Materials availability

Requests for plasmids generated in this study may be directed to Dr. Xin Wei Wang (xw3u@nih.gov).

#### Data and code availability

- scATAC-sequencing data generated from this manuscript is deposited with the Gene Expression Omnibus (GEO) under the accession GEO:GSE227265. This paper analyzes existing, publicly available data. These accession numbers for the datasets are listed in the key resources table. xCELLigence electrical impedance readings and original western blot images may be made available by contacting Dr. Xin Wei Wang.
- All original code is available in this paper’s supplemental information.
- Any additional information required to reanalyze the data reported in this work paper is available from the lead contact upon request.

### EXPERIMENTAL MODEL AND STUDY PARTICIPANT DETAILS

#### Human samples

The study included patients with HCC (n = 13) and iCCA (n = 3) enrolled into clinical trials at the National Cancer Institute (Bethesda, MD) and the University Medical Center in Mainz (Mainz, Germany). Mean age of patients at time of sample collection was 67 years with a slight male predominance (63%). Other patient characteristics were collected prospectively and reviewed as described in Table 1. Tissue was collected via tumor resection in 7 patients and needle biopsy in 9 patients. We collected samples from the tumor core. Each sample was measured about 5 mm diameter in size before single-cell library preparation. All patients provided written informed consent, and the study was approved by the National Institutes of Health Institutional Review Board and University Medical Center in Mainz.

#### Cell lines

Human CCA cell lines, HuCCT1 (JCRB0425) and HUH-28 (JCRB0426) were used. Cells were authenticated via Human Cell STR Profiling (ATCC) and tested negative for mycoplasma. Cells were cultured in Roswell Park Memorial Institute (RPMI) 1640 supplemented with 10% FBS (Invitrogen #12483020), 2mM L-glutamine solution (Gibco #25030) and 100 U/mL penicillin, and 100 μg/mL streptomycin (Gibco #15140-122) at 37°C in a humidified incubator with 5% CO2.

### METHOD DETAILS

#### Single cell suspension and nuclei extraction

Tissue dissociation was performed as previously described.^14,15^ Briefly, tissue was obtained in MACS Tissue Storage Solution (Miltenyi Biotech, Cat# 130-100-008) and immediately dissociated on ice. Tissue was first cut using a sterile scalpel into small pieces. The tissue was next places in a gentleMACS C Tube (Miltenyi Biotech, Cat#130-093-237) with 5 mL enzymatic digestion mix (Tumor Dissociation Kit from Miltenyi Biotech, Cat# 130-095-929) and homogenized using a gentleMACS Dissociator (Miltenyi Biotech). Dissociation was performed at 37°C for 30 min with 300 rpm shaking. The homogenized sample was further strained using a 70 μm cell strainer to remove undigested tissue (Miltenyi # 130-095-823). Single cells were flash frozen in liquid nitrogen and kept at −160°C until nuclei extractions were performed.

Nuclei isolation was performed following the 10X Genomics protocol (Nuclei isolation for single cell ATAC sequencing, revision D). Cells were briefly thawed in a 37°C water bath and resuspended in 10 mL RPMI media supplemented with 10% FBS. After centrifugation at 300 rcf for 5 min, the supernatant was removed, and the cell pellet was resuspended in 1 mL PBS supplemented with 0.04% BSA and passed through a 40 μm cell strainer. Sample concentration and viability were assessed using the LunaFL fluorescent cell counter. Cells were again centrifuged at 300 rcf for 5 min at 4°C and resuspended in 50 μL PBS +0.04% BSA. After an additional spin at 300 rcf for 5 min at 4°C, 45 μL of the supernatant was removed and 45 μL of chilled lysis buffer was added to the cell pellet. Cells were incubated in lysis buffer for 20 min on ice, after which 50 μL of chilled wash buffer was added, and cells were centrifuged at 500 rcf for 5 min at 4°C. 95 μL of the supernatant was removed, and nuclei were resuspended in 45μL of chilled 10X Genomics diluted nuclei buffer. After a final centrifugation at 500 rcf for 5 min at 4°C the supernatant was removed, and nuclei were resuspended in a final volume of 7 μL of chilled 10X Genomics diluted nuclei buffer. Sample concentration and viability were assessed using the LunaFL fluorescent cell counter.

#### Single cell ATAC data generation

Nuclei were loaded according to the 10X ATAC User Guide with a single capture lane per sample. Transposition and nuclei partitioning were completed successfully with uniform emulsion consistency and the GEM incubation PCR was run overnight. All subsequent steps of library preparation and quality control were performed as described in the 10X Genomics ATAC User Guide. 293–10,277 nuclei were captured per sample.

#### scATAC sequencing data processing

##### Sequencing

Sequencing of ATAC libraries was performed on an Illumina NovaSeq S2 instrument through the Center for Cancer Research Sequencing Facility. The run was as follows: 50bp (Read1), 8bp (Index1), 16bp (Index2), 50bp (Read2), 100 cycle run. 7,710–31,928 median fragments per nucleus were generated per sample.

##### FASTQ generation, read filtering and alignment

The standard 10X Genomics *cellranger-atac*, version 1.2.0, was used to extract FASTQs and 10X Genomics *cellranger-atac,* version 1.2.0, pipeline was used to perform data processing. Sequenced reads were aligned to the 10X Genomics provided human reference sequence (refdata-cellranger-atac-GRCh38–1.2.0). Demultiplexing and sample aggregation was performed using *cellranger* version 1.2.0.

##### Cell filtering and peak calling

Data processing was performed in concordance with the published Satpathy & Granja et al. pipeline (https://github.com/GreenleafLab/10x-scATAC-2019).^12^ Beginning with the fragment files generated from the *cellranger* pipeline, cells with less than 1,000 unique fragments were removed. Transcription start site (TSS) enrichment scores were calculated as previously described.^12^ TSS sites were acquired from the Bioconductor package ‘TxDb.Hsapiens.UCSC.hg38.knownGene’. Cells with a TSS enrichment score lower than 8 were also excluded from further analysis. Next, overlaps of Tn5 insertions with 2.5kb windows were found to create a cell by window sparse matrix. This matrix was binarized and filtered to include only the 20,000 most accessible sites. Normalization and dimension reduction (retaining top 50 dimensions) was performed using Latent Semantic Indexing (LSI), which includes calculating the term frequency-inverse document frequency (TF-IDF) transformation followed by singular value decomposition (SVD) using the Seurat R package v3.1.4.^35^ Initial graph-based clustering was also done using the Seurat package, including dimensions 2 to 25 and a resolution of .8x *N*, with *N* representing the number of iterations needed to create clusters of a minimum size of 200 cells. Dimension 1 was omitted as it reflected cell read depth. ATAC peaks were next called from the grouped cells from each cluster using the MACS2 callpeak command with parameters ‘–shift −75–extsize 150–nomodel–call-summits–nolambda–keep-dup all -q 0.05’.^41^ The peak summits were then extended by 150bp on either side, filtered by the ENCODE hg38 blacklist (https://sites.google.com/site/anshulkundaje/projects/blacklists) and then filtered to remove peaks that extended beyond the ends of chromosomes.^42^ To create a union peak set across all cells from all samples, an iterative process of finding overlapping peaks and discarding all but the most significant peak coordinates was performed, first across all cells within each cluster, and then across all cells from all samples. The generated binarized accessibility matrix was saved as an assay in a Seurat object.^35^ The fraction of reads in peaks (FRIP) was calculated using findOverlaps between the peaks included in the union peak set and all fragments, divided by the total number of fragments on a per cell basis. Further quality control was conducted using the Signac v0.2.5 R package.^13^ Signac functions TSSEnrichment, and NucleosomeSignal were used to generate TSS enrichment scores and nucleosome signal (ratio of fragments between 147bp and 294bp to fragments <145bp), respectively. Only cells with an FRIP >.15, TSS enrichment score >1.75 and Nucleosome Signal <10 were retained for further analysis.

##### Normalization and clustering

The top 5% most abundant peaks were identified using the Seurat function FindTopFeatures and used as input features for rerunning LSI on the binarized accessibility matrix (retaining top 50 dimensions).^35^ The Seurat functions FindNeighbors and FindClusters were used to identify nearest neighbor graph-based clusters using dimensions 1:50 as input features. Dimensions 1:50 were also used as input features for the Seurat function RunUMAP, which performs further dimension reduction for data visualization in 2-dimensional space. Upon subsetting the dataset to cell type specific populations, top abundant peak features were recalculated and used as input for further normalization and clustering as described above.

##### Peak annotation

To identify genomic regions that peaks were identified in such as promoter regions, introns and exons, peak annotation was performed using the ChIPSeeker R package using the annotatePeak function.^36^ The promoter region was designated as −3,000bp to +3,000bp from TSS. The transcript related object used for genomic region mapping was ‘TxDb.Hsapiens.UCSC.hg38.knownGene.’

##### Gene enrichment estimation

Gene enrichment scores were calculated using the Signac R package v1.0.0 using the GeneActivity function.^13^ The GeneActivity function computes counts per cell in gene body and promoter regions. ‘EnsDb.Hsapiens.v86’ was used for genomic region mapping and default settings were used for all other input parameters. Genes analyzed were limited to protein-coding only and promoter regions were designated as −2,000bp from TSS. Gene enrichment scores were further log normalized using the median counts across all genes as the scale factor.

##### Transcription factor motif enrichment estimation

Transcription factor (TF) motif enrichment scores were calculated using the ChromVAR R package.^17^ ChromVAR creates the TF motif enrichment scores by calculating deviations in chromatin accessibility at TF motif binding positions from the expected accessibility based on the average across all cells. Motif position frequency matrix for this analysis was obtained from JASPAR (https://jaspar.uio.no/).^43^ Peaks of each cell were annotated for the presence of TF motif presence using the CreateMotifMatrix function of the Signac R package.^13^ Implementation of ChromVAR was performed through the Signac function RunChromVAR.

##### Malignant TF motif enrichment analysis

Before malignant cell TF motif enrichment analysis, ChromVAR was rerun to calculate deviation in chromatin accessibility at motif binding positions specifically among malignant cells. To perform dimension reduction, principal components were calculated using the most variable TF motifs (variation >1.5) across malignant cells using the RunPCA function of the Seurat R package.^13^ The top 50 dimensions from this calculation were used as input features for the RunUMAP function. To decrease bias from sample H70, which had a substantially larger representation of malignant cells, cells from this sample were subset to the mean number of cells of other samples (N = 130) before variation calculations. Cells were randomly chosen, and the calculation was bootstrapped 10 times, retaining only the consensus variable TF motifs across all runs.

Patient enriched TF motif enrichment was calculated using the FindAllMarkers Seurat R package function using a Wilcox test.^13^ Differential TF motif enrichment between patients with HCC and iCCA was calculated from pseudobulk enrichment scores (average score across all cells with an added constant of .3) from each patient to avoid bias in cell number variation between patients. T-tests were run for each TF motif between patients with HCC and iCCA. P-values were adjusted using the Benjamini-Hochberg method. TF motifs were considered differentially enriched if they met the criteria of having an FDR <0.1 and a Log2FC > 0.2.

##### Cell type labeling

Matching scRNA-sequencing has previously been reported for all samples included in the scATAC-seq cohort and is publicly available (GSE125449 and GSE151530).^14,15^ Since cell type labeling has already been completed for these samples, we decided to use the Seurat R package FindTransferAnchors and TransferData functions to transfer labels from our scRNA-seq dataset to the scATAC-seq dataset.^13^ The scRNA-seq data was used as a reference to identify anchorsets from the scATAC-seq query gene enrichment estimation dataset. Canonical correlation analysis was run on both the reference RNA dataset and the scATAC gene enrichment estimation dataset. Features used during anchor calculation dimension reduction were the 2,000 most variable genes of the scRNA-seq dataset. The projected LSI was used for dimension reduction of weighted anchors for cell type label transfer. 98% of cells had a prediction score higher than 0.5.

Cell type specific peaks and TF motif enrichment were calculated using the FindAllMarkers Seurat R package function using logistic regression or Wilcox test.^13^ Nearest coding genes to peaks were found using the Signac R package function ClosestFeature and ‘EnsDb.Hsapiens.v86’ for genomic region mapping.^13^ T cell, B cell and monocyte subset populations were similarly annotated with subtype labels by transferring data from the scRNA-seq dataset. B cell subtypes were not previously labeled in the matching scRNA-seq dataset, so this was first done by identifying cluster enrichment for gene signatures of plasma cells, memory cells and naive B cells. Enrichment scores were assigned to each cell using the FindModuleScore Seurat R package function.^13^ Gene sets of B cell subtypes were identified from the PanglaoDB (https://www.panglaodb.se/index.html).

##### CNV estimation

CNV estimation was performed using the inferCNV R package.^39^ A slide window of 100 genes was used to calculate the average relative activity of genes to eliminate gene-specific patterns and to reflect the CNV. The CNV score is defined as the sum of the squared CNV profiles for each cell.

##### Trajectory analysis

Trajectory analysis was performed using the python package STREAM (single-cell trajectories reconstruction, exploration, and mapping).^40^ STREAM was specifically designed and tested to accommodate single cell epigenetic data, including scATAC-seq and single cell TF motif enrichment scores. TF motif enrichment estimation was performed as described above using the R package ChromVAR. Analysis was performed on each patient individually, using the patient cell by TF motif enrichment score matrix as input. Next, feature selection, principal component analysis and dimension reduction (using top 25 components) were performed. ElPiGraph was used for tree structure learning and fitting. The number of initial nodes used to calculate the minimum spanning tree was 20. For learning the elastic principal graph, R0, λ and μ parameters were set to default values. Further optimization was performed on the elastic principal graph using the extend_elastic_principal_graph function with the epg_ext_mode parameter set to “WeightedCentroid” and the epg_ext_par parameter set to 0.8.

#### scRNA sequencing data generation and data processing

scRNA sequencing data has been previously reported.^14,15^ Data is deposited at the Gene Expression Omnibus (GEO) public data-base at NCBI (GEO:GSE125449 and GEO:GSE151530). Briefly, single-cell cDNA libraries were constructed following the 10x Genomics Single Cell 3′ Reagent Kit v2 or v3 user guide. Sequencing was performed using the Illumina HiSeq 4000/NovaSeq 6000 system with parameters of 26/28 cycles for barcodes, 8 cycles for sample indices and 98 cycles for cDNA reads. Demultiplexing was performed using bcl2fastq, with one mismatch allowed in the barcodes. The 10X Genomics Cell Ranger software was further applied for alignment, tagging, gene and transcript counting. Further downstream data processing was performed using the R package Seurat, including data normalization, scaling, clustering and UMAP dimension reduction.^13^

#### Bulk RNA sequencing cohorts

The TIGER-LC Cohort expression data is deposited at the GEO public database at NCBI (GEO:GSE76297).^25^ Figures from the Thailand Initiative in Genomics and Expression Research for Liver Cancer (TIGER-LC) Cohort were constructed from log normalized intensity expression values. The Cancer Genome Atlas Liver Hepatocellular Carcinoma (TCGA-LIHC) data was downloaded from the National Cancer Institute’s GDC Data Portal (https://portal.gdc.cancer.gov/). Matched clinical data were downloaded from the cBio-Portal (http://www.cbioportal.org/). An additional intrahepatic cholangiocarcinoma expression dataset was downloaded from the International Cancer Genome Consortium (ICGC) database (https://dcc.icgc.org).^33^

#### Gene set enrichment analysis, TF enrichment score calculations and molecular classification assignment

Gene set enrichment scores were calculated for each cell of the scATAC-seq cohort using the AddModuleScore function of the Seurat R package.^13^ The number of control features used for these calculations was 5. The AddModuleScore function calculates the average enrichment score across all features included in the gene or TF sets, normalized to the control features. Cell type specific gene signatures are listed. To identify gene sets enriched specifically within each cell type, differential gene enrichment was identified as described above. Identified genes were read in as features to the R package ClusterProfiler enricher function, which returned a list of gene sets that included these genes from the Molecular signatures database cell type signature gene sets collection.^44^

TF motif enrichment scores were generated for bulk RNA-seq samples of the TIGER-LC, TCGA and ICGC cohorts using the GSVA R package with the method parameter selected for ssGSEA.^21^ The 31 TF motif signature was generated by identifying significantly differentially enriched motifs in the scATAC-seq cohort and are listed in Table S5. TF target gene sets were downloaded from the TRRUST database.^45^ TIGER-LC samples were assigned previously published molecular classifications using Nearest Template Prediction (NTP) implemented with R.^46^

#### Generation of stable POU2F1-KO cell using CRISPR/Cas9 genome editing

Two distinct sgRNAs targeting *POU2F1* were designed at the NIH Genomics Core: pCam37682-POU2F1-562 (target sequence: GTAAACTTGTTCCAGCGAGTTGG) and pCam27683-POU2F1-563 (target sequence: GCACCAACACAAACTGTTGCAGG). One non-targeting sgRNA was also designed (pCam37686-NTC-GFP-686) to be used as a control. Lenti-Cas9-Blast (Addgene #52962) was purchased from Addgene and used to express Cas9.

Competent bacteria in glycerol were amplified in LB (Lennox) broth. DNA was purified using the Monarch Plasmid DNA MiniPrep (Monarch #T1010L) and QIAGEN Plasmid Midi Kit (QIAGEN #12145). DNA was transfected in HEK293T cells using Lenti-Pac HIV Expression Packaging Kit (GeneCopoeia #LT002). HuCCT1 and HUH-28 cells were transduced with a combination of LentiCas9-Blast and pCam37686-NTC-GFP-686 (as non-target vector), LentiCas9-Blast and pCam37682-POU2F1-562 or pCam27683-POU2F1-563 (as POU2F1-KO) viral particles supplemented with 8 μg/mL polybrene for 48 h and selected using 10 μg/mL of Blasticidin and 1 μg/mL puromycin for >10 days. Validation of stable-cell lines was performed by checking GFP (pCam37686-NTC-GFP-686) and RFP (pCam37682-POU2F1-562 and pCam27683-POU2F1-563) signals at a fluorescence microscope and immunoblotting for anti-POU2F1, whereas anti-β-actin was included as control.

#### Generation of single cell-derived clones

Stable HuCCT1 and HUH-28 transduced cells were digested into single cells and plated in 96-well plates containing medium. Validation of single clones was performed by checking GFP (pCam37686-NTC-GFP-686) and RFP (pCam37682-POU2F1-562 and pCam27683-POU2F1-563) signals at a fluorescence microscope on the next day and for the coming weeks. POU2F1 knockout was validated by immunoblotting for anti-POU2F1 and only clones with no protein expression left were selected for further investigation, named as: HuCCT1-NTC-5, HuCCT1-POU2F1-562-5, HuCCT1-POU2F1-563-4, HUH-28-NTC-6, HUH-28-POU2F1-562-2 and HUH-28-POU2F1-562-4.

#### *In vitro* cell migration

The xCELLigence RTCA DP biosensor system (Agilent) was utilized to compare the *in vitro* migration rates of HuCCT1 and HUH-28 cells after POU2F1-KO. In brief, 50,000 cells were seeded to each well of CIM-Plate 16-well (Agilent #5665817001) and the electrical impedance created in each well were monitored for 24 h. Each experiment was performed three times in quadruplicate wells. The changes of impedance were displayed as Cell Index.

#### Western blot

Cells were harvested in PBS and homogenized in RIPA buffer (ThermoFisher Scientific #89900) supplemented with protease inhibitors (Takara #ST0293), for 10 min at 4°C before centrifugation at 12,000 g at 4°C for 20 min. Proteins were quantified using the Pierce BCA Protein Assay kit (ThermoFisher Scientific #23225). Total protein extract between 30 and 80 μg were loaded in a NuPAGE 4–12%, Bis-Tris, 1.5mm Mini Protein Gel (Bio-Rad #NP0321BOX). Transfer into a PVDF membrane was performed using the TRANS-Blot Turbo RTA Transfer Kit (Bio-Rad #1704272). After transfer, membranes were blocked with a 5% solution of nonfat powdered milk (LabScientific #732-291-1940) in PBS 0.1% Tween for a minimum of 1 h before antibody hybridization. The following primary antibodies were incubated overnight at 4°C: POU2F1 (1:1000) (Abcam #ab178869) and β-actin (1:1000) (Abcam #ab6276). HRP Anti-rabbit (Millipore Sigma #NA934v) and HRP Anti-mouse (Millipore Sigma #NA931v) were used as secondary antibodies for detection using luminescence (Clarity Max Western ECL Blotting Substrates (Biorad #1705062). Images were captured using a Chemidoc MP Image System (Bio-Rad) and cropped using the Image Lab Software. Experiments were repeated using total protein extract from biological triplicates.

#### Clonogenic assay of colony formations

1,000 cells were seeded in 6-well plates containing medium and incubated for 10 days at 37°C in a humidified incubator with 5% CO2. Each well was washed twice with PBS and fixed with ice-cold Methanol for 15 min before staining with 0.5% Crystal violet for 2 h. Distilled water was used to carefully rinse the wells. Plates were left at room temperature to dry. Images were captured using a Chemidoc MP Image System (Bio-Rad) in the Colorimetric function. Colonies were manually counted. 3 biological experiments were performed.

#### Graph generation

To visualize ATAC peaks across a genomic region of interest, we used the CoveragePlot function of the Signac R package.^13^ Gene coordinates for annotation were extracted from ‘EnsDb.Hsapiens.v86’ using the UCSC naming convention. Pie chart of peaks within known genomic regions was created using ChIPSeeker plotAnnoPie function.^36^ UMAP projections were created using the UMAPPlot function of the Signac R package.^13^ When visualizing continuous variables such as gene or TF enrichment scores, the FeaturePlot function was used, capping the max cut off at q90 and min cut off at q10. Hierarchical clustering and heatmap generation was performed using the R package ComplexHeatMap using a Pearson correlation distance matrix.^38^ Heatmap generation of scATAC-seq data was performed using the Seurat R package DoHeatmapFunction with default parameters. Violin plots were generated using the Seurat R package VlnPlot with default parameters.^13^ Bar graphs and boxplots were generated using the R package ggplot2.^37^ For each boxplot, the center line represents the median. Upper and lower limits of each box represent the 75th and 25th percentiles, respectively. The whiskers represent the lowest data point still within 1.5 × box size of the lower quartile and the highest data point still within 1.5 × box size of the upper quartile. Interaction network between differentially enriched TF motifs was generated and visualized using QIAGEN Ingenuity Pathway Analysis. Flat trajectory tree plots were created using the python package STREAM.^40^

### QUANTIFICATION AND STATISTICAL ANALYSIS

Using the Graph Pad software (Prism, version 9.2), two-way ANOVA was performed for the *in vitro* cell migration experiments and one-way ANOVA was performed for the clonogenic assay of colony formations. T-tests, one-way ANOVA, logistic regression, Pearson’s Chi-Squared, Spearman’s rank correlation and Wilcoxon tests were performed using R to determine p values. Kruskal–Wallis H-test followed by post-hoc pairwise Conover’s test was used to determine branch enriched TF motif enrichment after tree fitting for trajectory analysis (Table S8). Kaplan-Meier survival analysis was performed using R with the packages Survival and Survminer. The log rank test was used to determine statistical significance. NR high groups in the TIGER-LC and TCGA HCC cohorts were defined as patients with NR enrichment scores in the highest quartile. The POU low group in the TIGER-LC iCCA cohort was defined as patients with POU enrichment scores in the lowest quartile. The POU high group in the ICGC iCCA cohort was defined as patients with POU enrichment scores in the highest quartile. For testing the 31 TF motif signature as a tumor subtype classifier, TIGER-LC data was divided into a training (80%) and a test set (20%). Using 5-fold cross-validation logistic regression, the 31 TF motif signature was regressed on tumor status (HCC vs. iCCA) using ROSE oversampling to account for the class imbalance in the training set.^47^ A receiver operating curve (ROC) was generated representing the performance of the model in te test data.

## Supplementary Material

1

2

3

4

5

6

7

8

9

10

## Figures and Tables

**Table:** KEY RESOURCES TABLE

REAGENT or RESOURCE	SOURCE	IDENTIFIER
Antibodies		
Anti-POU2F1 (OCT-1)	Abcam	#Ab178869
Anti-B-actin	Abcam	Ab6276;RRID:AB_2223210
HRP Anti-Rabbit	Millipore Sigma	#NA934v
HRP Anti-Mouse	Millipore Sigma	#NA931v
Bacterial and virus strains		
LentiCas9-Blast	Addgene	#52962
pCam37682-POU2F1-562	This paper	NIH Genomics Core
pCam27683-POU2F1-563	This paper	NIH Genomics Core
Chemicals, peptides, and recombinant proteins		
Clarity Max Western ECL Blotting Substrates	Biorad	#170562
RPMI 1640 medium	Gibco	#11875093
Fetal Bovine Serum	Fisher Scientific	#SH30396.03
Pierce Bovine Serum Albumin Standard Ampules	ThermoFisher Scientific	#23209
Pierce RIPA Buffer	ThermoFisher Scientific	#89900
Protease Inhibitors Cocktail (100x)	Takara	#ST0293
Puromycin [10 mg/mL]	ThermoFisher Scientific	#J67236.8EQ
Blasticidin S HCl [10 mg/mL]	ThermoFisher Scientific	#A1113903
Polybrene	Sigma-Aldrich	#TR-1003-G
LB (Lennox) Borth	KD Medical	#BLE-3060
Penicilin-Streptomycin	Gibco	#15140-122
L-glutamine 200mM (100x)	Gibco	#25030-081
Non-Fat Dry Milk	LabScientific	#732-291-1940
Critical commercial assays		
Chromium Next GEM Single Cell ATAC Library & Gel Bead Kit	10x Genomics	PN-1000175
Lenti-Pac HIV Expression Packaging kit	Genecopoeia	#LT002
Pierce BCA Protein Assay kit	ThermoFisher Scientific	#23225
QIAGEN Plasmid Midi kit	QIAGEN	#12145
Monarch Plasmid DNA MiniPrep kit	Monarch	#T1010L
TRANS-Blot Turbo RTA Transfer Kit, PVDF	Bio-Rad	#1704272
Deposited data		
TIGER-LC Cohort expression	Chaisaingmongkol et al.^25^	GEO:GSE76297
The Cancer Genome Atlas Liver Hepatocellular Carcinoma	National Cancer Institute’s GDC Data Portal	https://portal.gdc.cancer.gov/
International Cancer Genome Consortium database	International Cancer Genome Consortium Portal	https://dcc.icgc.org
scRNA-seq 1	Ma et al.^15^	GEO:GSE125449
scRNA-seq 2	Ma et al.^14^	GEO:GSE151530
scATAC-sequencing	This paper	GEO:GSE227265
Experimental models: Cell lines		
HuCCT1	JCRB Cell Bank	JCRB0425
HUH28	JCRB Cell Bank	JCRB0426
Lenti-Pac 293Ta cell line	GeneCopoeia	#LT008
Oligonucleotides		
pCam37682-POU2F1-562 (GTAAACTTGTTCCAGCGAGTTGG)	This paper	NIH Genome Modification Core
pCam27683-POU2F1-563 (GCACCAACACAAACTGTTGCAGG)	This paper	NIH Genome Modification Core
Software and algorithms		
Cellranger-atac (version 1.2.0)	Satpathy et al.^12^	N/A
Seurat R package v3.1.4	Stuart et al.^35^	N/A
ChIPSeeker R package	Yu et al.^36^	N/A
Signac R package v1.0.0	Stuart et al.^13^	N/A
ChromVAR R Package	Schep et al.^17^	N/A
Survival R package	CRAN:Package survival (r-project.org)	N/A
Survminer R package	CRAN:Package survminer (r-project.org)	N/A
ggplot2 R package	Wickman^37^	N/A
ComplexHeatMap R package	Gu et al.^38^	N/A
GSVA R package	Hanzelmann et al.^21^	N/A
MACS2	MACS2 - Docs CSC	N/A
GraphPad	Home - GraphPad	N/A
QIAGEN Ingenuity Pathway Analysis	QIAGEN Ingenuity Pathway Analysis (IPA), https://www.qiagen.com/us/products/discovery-and-translational-research/next-generation-sequencing/informatics-and-data/interpretation-content-databases/ingenuity-pathway-analysis	830018
PanglaoDB	https://www.panglaodb.se/index.html	N/A
inferCNV R package	Patel et al.^39^	N/A
STREAM python package	Chen et al.^40^	N/A
Biorender	Scientific Image and Illustration Software | BioRender, https://www.biorender.com/	N/A
Other		
MACS Tissue Storage Solution	Miltenyi Biotech	#130-100-008
gentleMACS C Tube	Miltenyi Biotech	#130-093-237
Tumor Dissociation Kit – Enzymatic Digestion Mix	Miltenyi Biotech	#130-095-929
70 mm cell strainer	Miltenyi Biotech	#130-095-823
CIM Plate 16-well	Agilent	#5665817001
NuPAGE 4–12%, Bis-Tris, 1.5mm Mini Protein Gel	Invitrogen	#NP0321BOX

**Table 1.** Patient demographics and tumor characteristics

Sample	Diagnosis	Age	Gender	Race	Stage	Etiology	Biopsy timing	Immunotherapy treatment	Local therapy	scATAC cells	scRNA cells	Grade	Driver mutations
H38	HCC	74	male	Asian	I	none	pre-resection	none	resection	1,803	1,037	moderately differentiated	–
H62	HCC	67	female	White	I	HCV	pre-resection	none	resection	1,852	519	well differentiated	–
H70	HCC	72	male	White	II	autoimmune hepatitis	pre-resection	none	resection	5,069	7,205	undifferentiated	NFE2L2 TSC2 TP53 ROS1
1HT1	HCC	70	male	White	II	metabolic disease	pre-resection	none	resection	2,201	1,509	poorly differentiated	–
2HT1	HCC	77	female	–	I	none	pre-resection	nivolumab	resection	2,097	7,141	moderately differentiated	–
4HT1	HCC	76	male	Black	I	HCV, HIV	pre-resection	none	resection	1,296	1,450	undifferentiated	–
H77	HCC	72	male	White	III	HCV	pre-I/O start	tremelimumab/durvalumab	TACE	472	4,800	poorly differentiated	PBRM1 TSC1
H30	HCC	63	male	White	IV	HCV	pre-I/O start	tremelimumab/durvalumab	XRT (pancreas)	706	787	undifferentiated	AHCTF1 ERBB2 IL6ST TP53
H65	HCC	62	female	White	IV	HCV	pre-I/O start	tremelimumab/durvalumab	none	216	594	–	CDKN2A RB1
H58	HCC	73	male	Hispanic	IV	EtOH	1 day after I/O start	tremelimumab/durvalumab	TACE	274	1,244	moderately differentiated	ARID1A
H63	HCC	81	male	Asian	IV	HBV	pre-I/O start	tremelimumab/durvalumab	ablation	279	545	poorly differentiated	ARID1A
H23	HCC	42	female	White	IV	NASH	1 day after I/O start	tremelimumab/durvalumab	TACE	148	88	moderately differentiated	BAP1 GNAS NF1
H69	HCC	58	male	White	–	HCV	pre-I/O start	tremelimumab/durvalumab	TACE	409	3,193	poorly differentiated	CDKN1A
C60	iCCA	79	female	White	III	none	pre-resection	none	resection	839	1,440	moderately differentiated	IDH1 APC
C56	iCCA	52	female	White	IV	none	pre-I/O start	tremelimumab/durvalumab	ablation	143	137	poorly differentiated	ARAF BAP1 NOTCH3
C39	iCCA	61	male	White	IV	none	1 day after I/O start	pembrolizumab	none	805	438	poorly differentiated	ARID1B PTEN RPS6KA3

## Highlights

- scATAC-seq of 16 patients reflects the compositional diversity of liver cancer
- Transcription factor motif enrichment discriminates HCC from iCCA
- The POU motif family in iCCA tumors is associated with poor prognosis in iCCA
