# Supplementary material for: Genome-wide profiling of transcription factor activity in primary liver cancer using single-cell ATAC sequencing
Source: Cell Rep. Author manuscript; Available in PMC 2023 Dec 26. (PMC10750269; doi:10.1016/j.celrep.2023.113446)
Supplement: 1 [file NIHMS1948211-supplement-1.pdf]

Cell Reports, Volume *42*

Supplemental information

Genome-wide profiling of transcription factor activity in primary liver cancer using single-cell ATAC sequencing

Amanda J. Craig, Maruhen A. Datsch Silveira, Lichun Ma, Mahler Revsine, Limin Wang, Sophia Heinrich, Zachary Rae, Allison Ruchinskas, Kimia Dadkhah, Whitney Do, Shay Behrens, Farid R. Mehrabadi, Dana A. Dominguez, Marshonna Forgues, Anuradha Budhu, Jittiporn Chaisaingmongkol, Jonathan M. Hernandez, Jeremy L. Davis, Bao Tran, Jens U. Marquardt, Mathuros Ruchirawat, Michael Kelly, Tim F. Greten, and Xin W. Wang

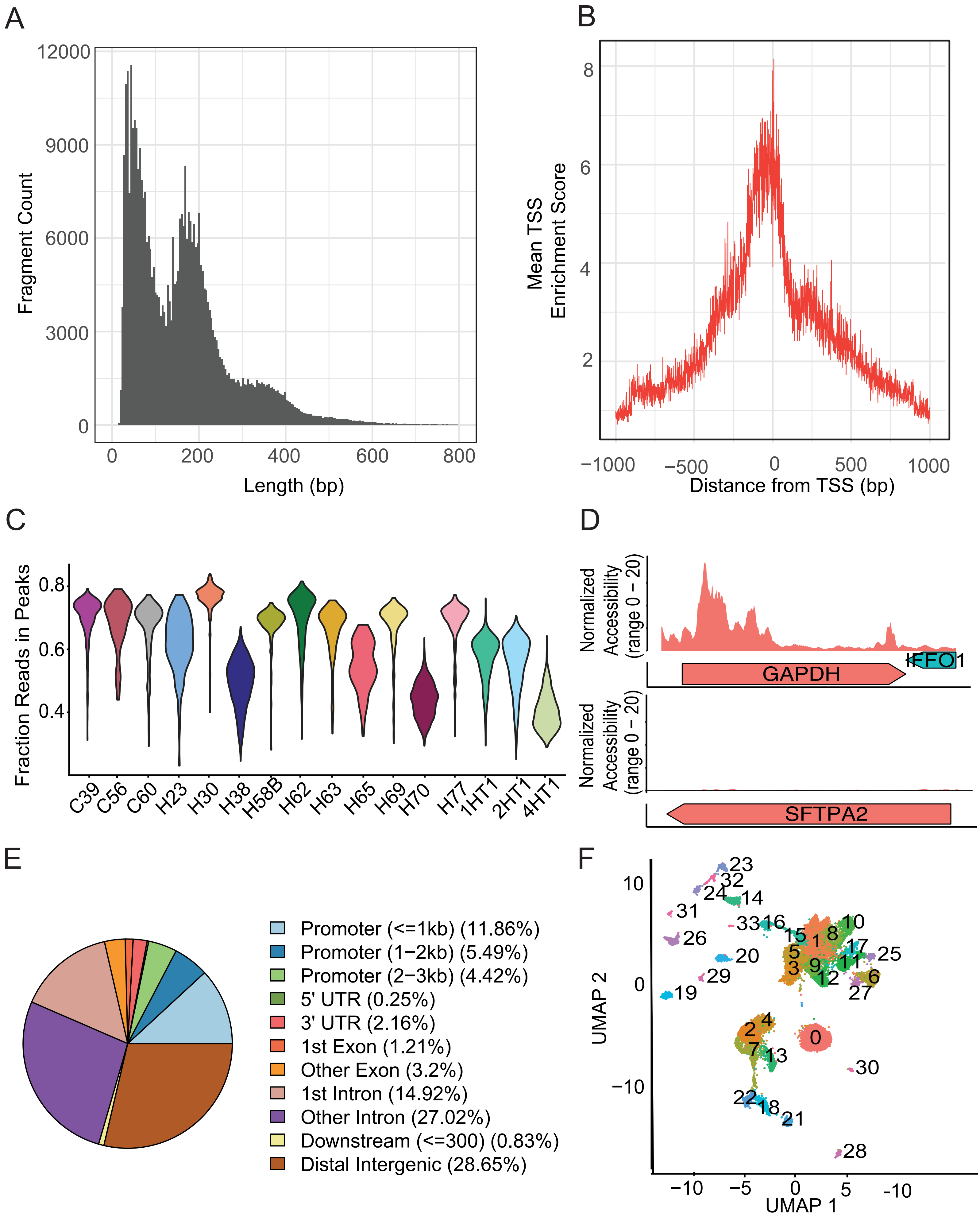

A
Fragment Count
Length (bp)
B
Mean TSS Enrichment Score
Distance from TSS (bp)
C
Fraction Reads in Peaks
C39 C56 C60 H23 H30 H38 H58B H62 H63 H65 H69 H70 H77 1HT1 2HT1 4HT1
D
Normalized Accessibility (range 0 − 20)
GAPDH
IFFO1
SFTPA2
E
Promoter (<=1kb) (11.86%)
Promoter (1−2kb) (5.49%)
Promoter (2−3kb) (4.42%)
5' UTR (0.25%)
3' UTR (2.16%)
1st Exon (1.21%)
Other Exon (3.2%)
1st Intron (14.92%)
Other Intron (27.02%)
Downstream (<=300) (0.83%)
Distal Intergenic (28.65%)
F
UMAP 2
UMAP 1

Supplemental Figure 1 – scATAC-seq metrics

A. Periodicity histogram showing lengths of fragments.

B. Average transcription start sites enrichment scores at different distances from transcription start sites.

C. Violin plots of fraction of reads in peaks by patients.

D. GAPDH and SFTPA2 gene tracks.

E. Pie chart of peak location annotations.

F. UMAP visualization of all 18,609 cells. Each color represents a graph-based cluster.

Related to Figure 1.

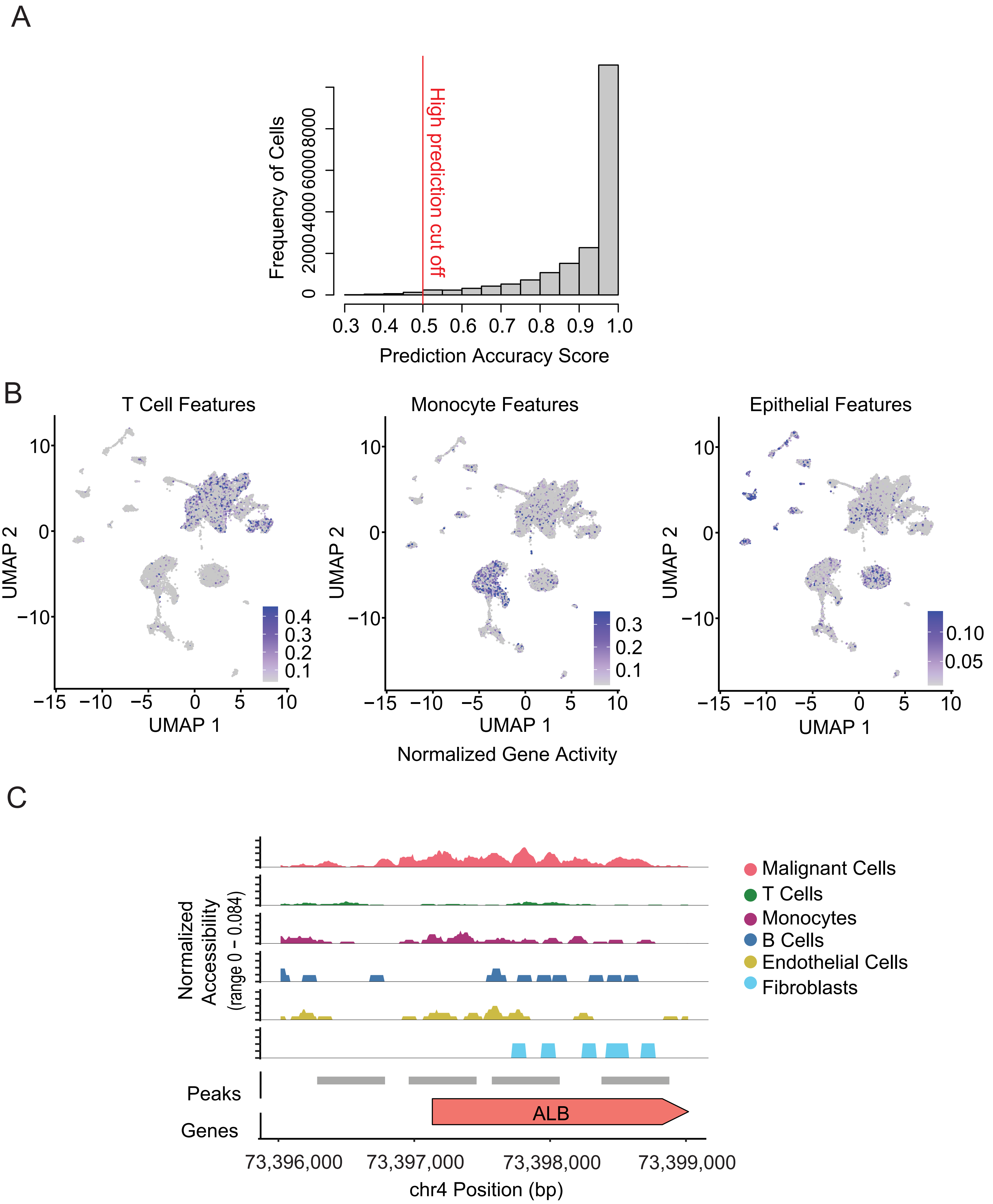

A
Frequency of Cells
0
2000
4000
6000
8000
High prediction cut off
0.3 0.4 0.5 0.6 0.7 0.8 0.9 1.0
Prediction Accuracy Score
B
T Cell Features
Monocyte Features
Epithelial Features
UMAP 2
UMAP 1
10
0
−10
−15 −10 −5 0 5 10
0.4
0.3
0.2
0.1
0.10
0.05
Normalized Gene Activity
C
Normalized Accessibility (range 0 – 0.084)
Malignant Cells
T Cells
Monocytes
B Cells
Endothelial Cells
Fibroblasts
Peaks
Genes
ALB
73,396,000
73,397,000
73,398,000
73,399,000
chr4 Position (bp)

Supplemental Figure 2 – scATAC-seq cell type annotation

A. Label transfer prediction score histrogram.

B. UMAP visualization of all 18,609 cells. Color represents cell type enrichment scores.

C. ALB gene track. Each color represents peaks from different cell types.

Related to Figure 1.

A

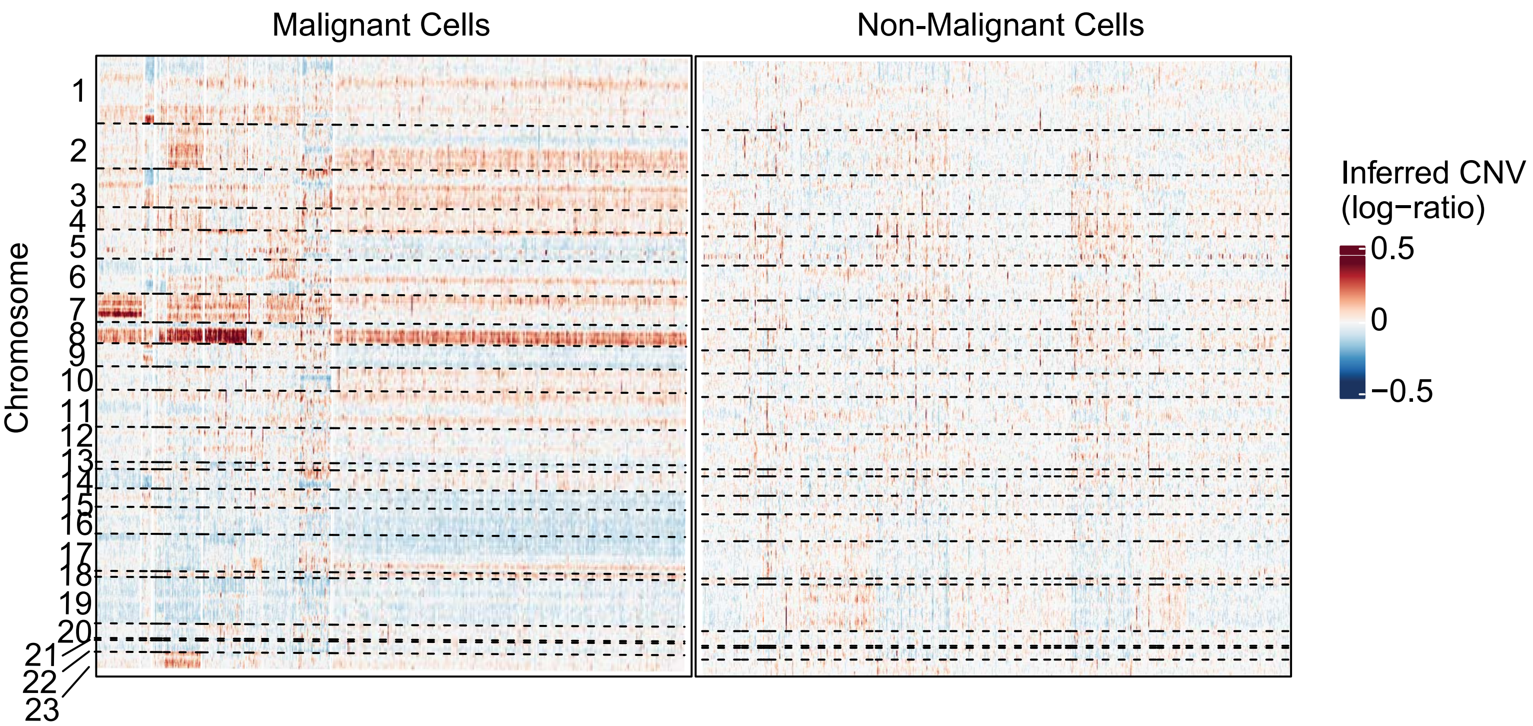

Malignant Cells
Non-Malignant Cells
Chromosome
1
2
3
4
5
6
7
8
9
10
11
12
13
14
15
16
17
18
19
20
21
22
23
Inferred CNV
(log−ratio)
0.5
0
−0.5

B

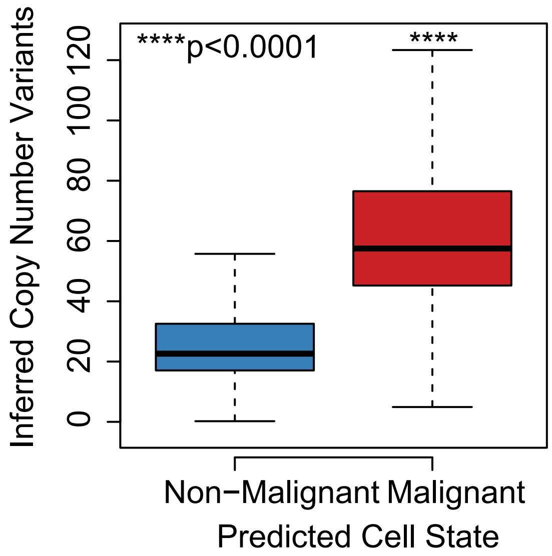

****p<0.0001
****
Inferred Copy Number Variants
0
20
40
60
80
100
120
Non−Malignant
Malignant
Predicted Cell State

Supplemental Figure 3 – Identification of malignant cells by scATAC-seq

A. Heatmap of inferred copy number variants.

B. Boxplot of inferred copy number variants in predicted non-malignant and malignant cells. For each boxplot, the center line represents the median. Upper and lower limits of each box represent the 75th and 25th percentiles, respectively.

Related to Figure 1

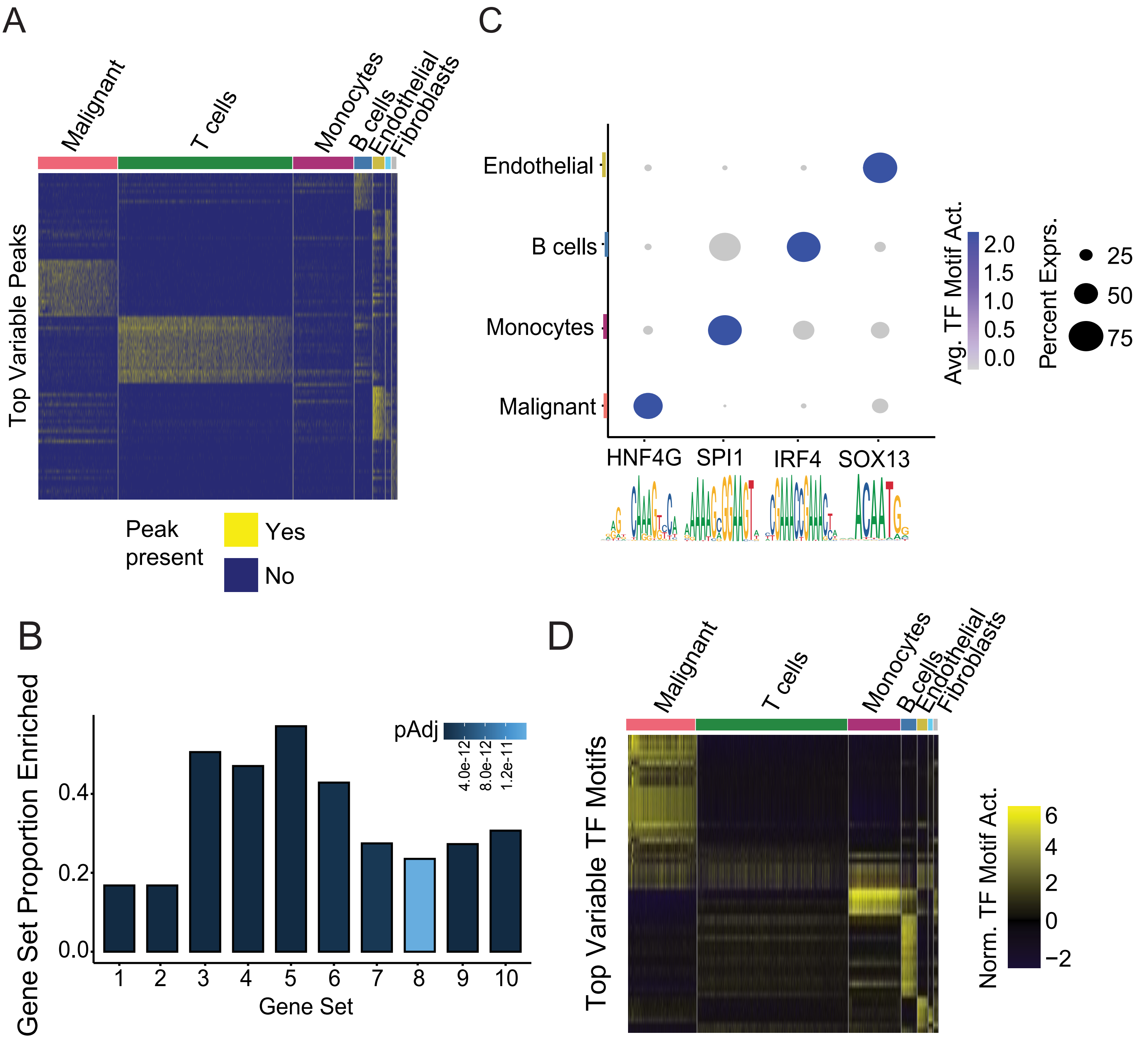

Gene Set:	Enriched cells:
1. Aizarani Liver C33 stellate cells-2	Fibroblasts
2. Lake adult kidney c27 vascular smooth muscle cells and pericytes	Fibroblasts
3. Aizarani Liver C10 MVECS-1	Endothelial cells
4. Muraro Pancreas Endothelial cell	Endothelial cells
5. Travaglini Lung B-cell	B cells
6. Hay Bone marrow follicular B-cell	B cells
7. Travaglini lung classical Monocyte cell	Monocytes
8. Aizarani Liver C2 Kupfer cells-1	TAMs
9. Aizarani Liver C28 NK NKT cells-6	T cells
10. Aizarani Liver C3 NK NKT Cells-2	T cells

Supplemental Figure 4 – Cell type enrichment

A. Heatmap of open peak markers for each cell type.

B. Gene set enrichment proportions for cell type signature gene sets for each cell type. Gene set enrichment analysis was assessed using closest genes to open peak markers for each cell type.

C. Dot plot showing TF motif activity markers for each cell type.

D. Heatmap of TF motif activity markers for each cell type.

Related to Figure 1

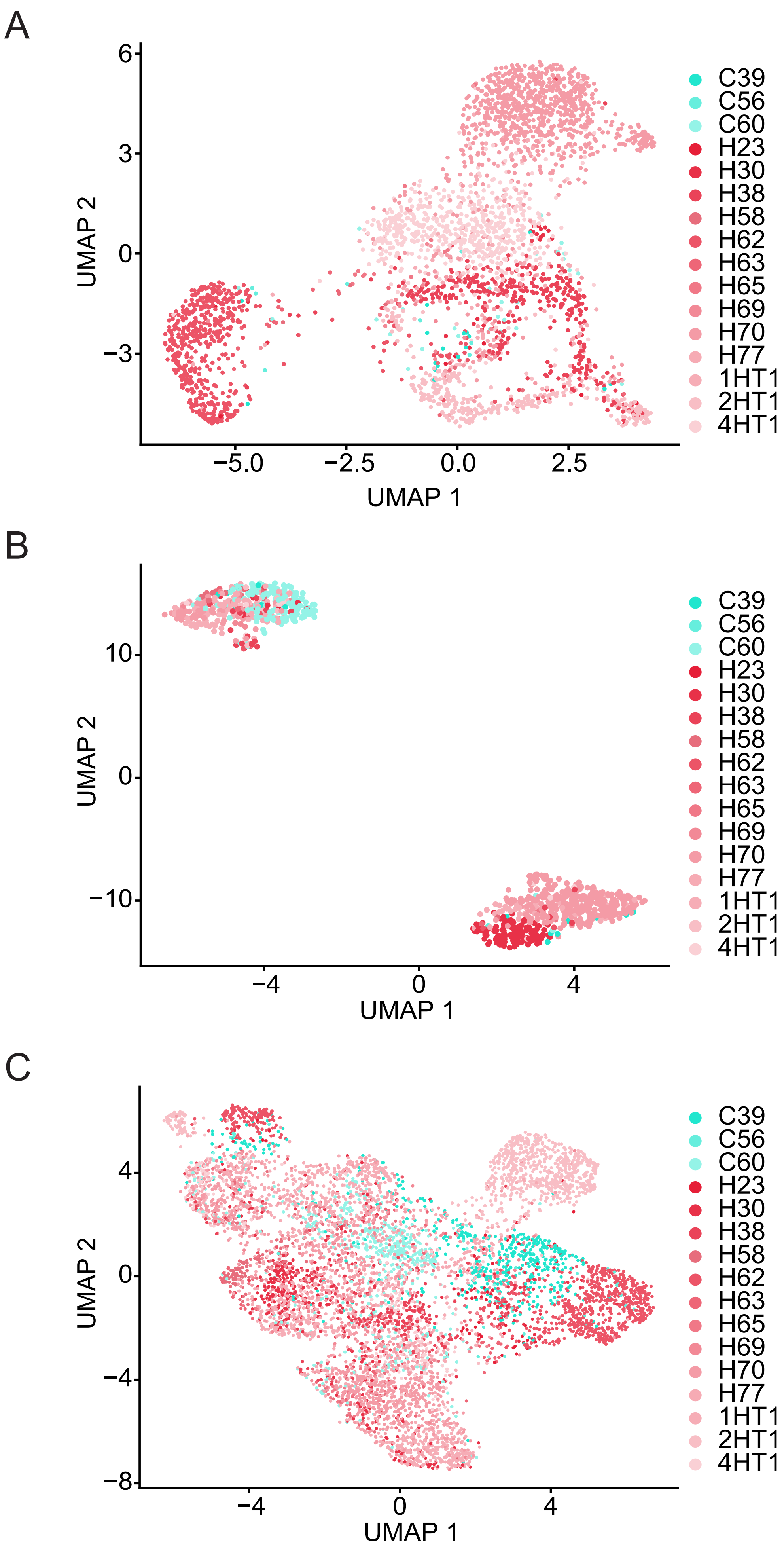
A
UMAP 2
6
3
0
−3
−5.0
−2.5
0.0
2.5
UMAP 1
C39
C56
C60
H23
H30
H38
H58
H62
H63
H65
H69
H70
H77
1HT1
2HT1
4HT1
B
UMAP 2
10
0
−10
−4
0
4
UMAP 1
C39
C56
C60
H23
H30
H38
H58
H62
H63
H65
H69
H70
H77
1HT1
2HT1
4HT1
C
UMAP 2
4
0
−4
−8
−4
0
4
UMAP 1
C39
C56
C60
H23
H30
H38
H58
H62
H63
H65
H69
H70
H77
1HT1
2HT1
4HT1

Supplemental Figure 5 – Immune cell clustering

A. UMAP visualization of monocytes.
B. UMAP visualization of B cells.
C. UMAP visualization of T cells.

Each color represents a patient. Blue shades are iCCA and pink shades are HCC.
Related to Figure 2

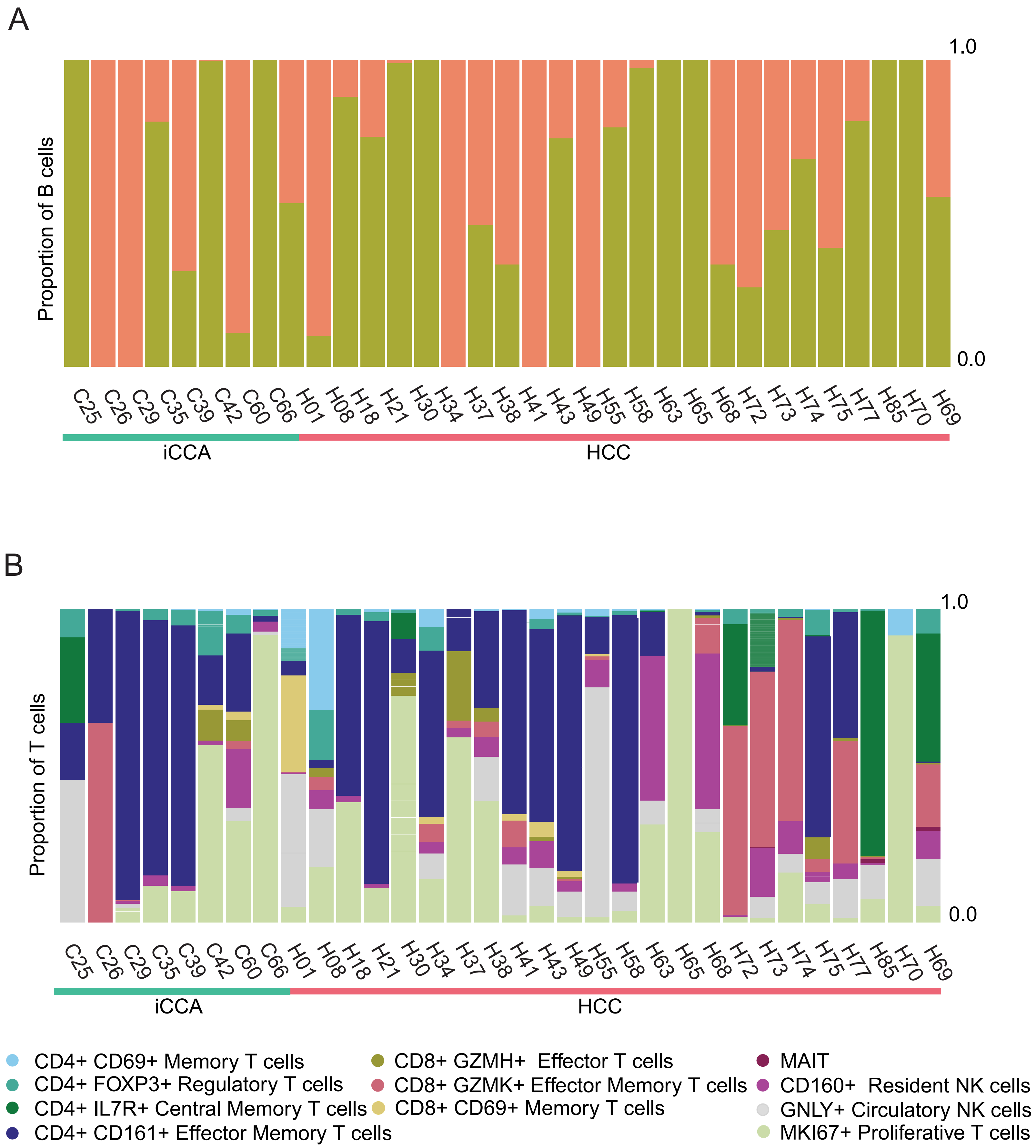
A
1.0
0.0
Proportion of B cells
C25 C26 C29 C35 C39 C42 C60 C66 H01 H08 H18 H21 H30 H34 H37 H38 H41 H43 H49 H55 H58 H63 H65 H68 H72 H73 H74 H75 H77 H85 H70 H69
iCCA
HCC
B
1.0
0.0
Proportion of T cells
C25 C26 C29 C35 C39 C42 C60 C66 H01 H08 H18 H21 H30 H34 H37 H38 H41 H43 H49 H55 H58 H63 H65 H68 H72 H73 H74 H75 H77 H85 H70 H69
iCCA
HCC
CD4+ CD69+ Memory T cells
CD4+ FOXP3+ Regulatory T cells
CD4+ IL7R+ Central Memory T cells
CD4+ CD161+ Effector Memory T cells
CD8+ GZMH+ Effector T cells
CD8+ GZMK+ Effector Memory T cells
CD8+ CD69+ Memory T cells
MAIT
CD160+ Resident NK cells
GNLY+ Circulatory NK cells
MKI67+ Proliferative T cells

Supplemental Figure 6 – Tumor microenvironment composition by patient

A. Relative abundance of B cell subtypes in each scRNA-seq patient.

B. Relative abundance of T cell subtypes in each scRNA-seq patient.

Related to Figure 2

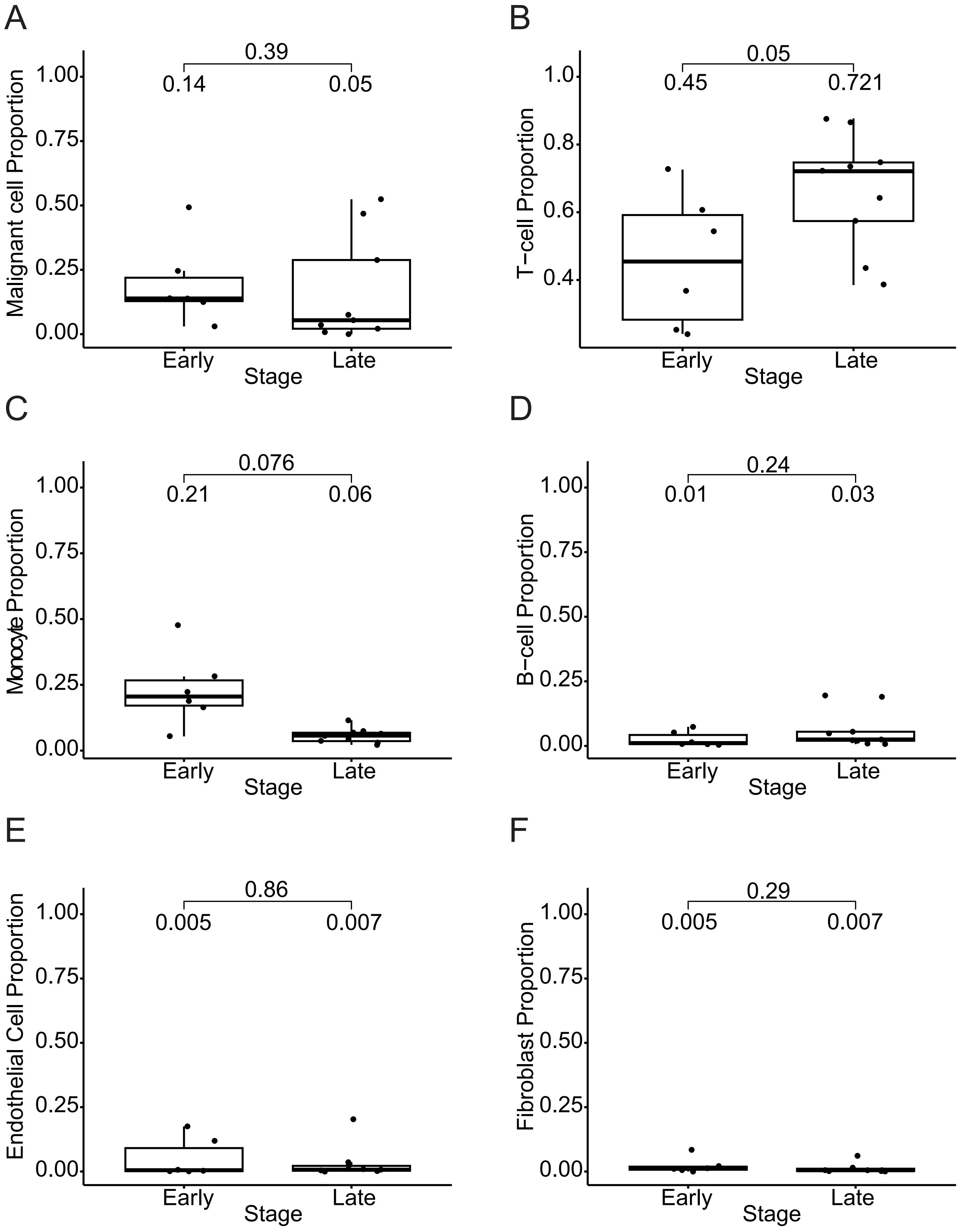
A
0.39
0.14
0.05
Malignant cell Proportion
1.00
0.75
0.50
0.25
0.00
Early
Late
Stage
B
0.05
0.45
0.721
T−cell Proportion
1.0
0.8
0.6
0.4
Early
Late
Stage
C
0.076
0.21
0.06
Monocyte Proportion
1.00
0.75
0.50
0.25
0.00
Early
Late
Stage
D
0.24
0.01
0.03
B−cell Proportion
1.00
0.75
0.50
0.25
0.00
Early
Late
Stage
E
0.86
0.005
0.007
Endothelial Cell Proportion
1.00
0.75
0.50
0.25
0.00
Early
Late
Stage
F
0.29
0.005
0.007
Fibroblast Proportion
1.00
0.75
0.50
0.25
0.00
Early
Late
Stage

Supplemental Figure 7 – Tumor microenvironment composition by tumor stage

A. Boxplots of proportion of total cells detected classified as malignant cells comparing early to late stage tumors.

B. Boxplots of proportion of total cells detected classified as T cells comparing early to late stage tumors.

C. Boxplots of proportion of total cells detected classified as monocytes comparing early to late stage tumors.

D. Boxplots of proportion of total cells detected classified as B cells comparing early to late stage tumors.

E. Boxplots of proportion of total cells detected classified as endothelial comparing early to late stage tumors.

F. Boxplots of proportion of total cells detected classified as fibroblasts comparing early to late stage tumors.

For each boxplot, the center line represents the median. Upper and lower limits of each box represent the 75th and 25th percentiles, respectively.

Related to Figure 2

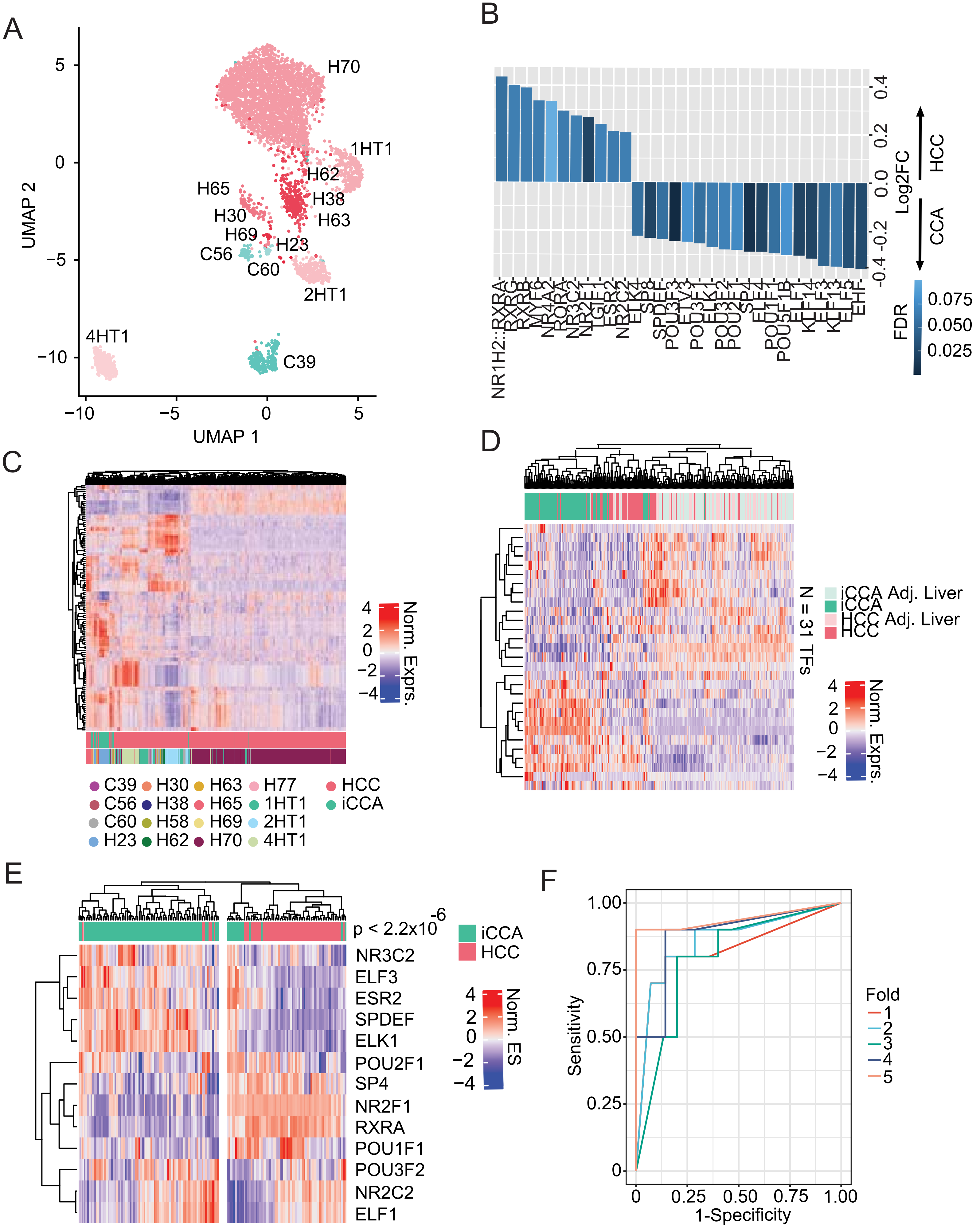

A
B
C
D
E
F
UMAP 1
UMAP 2
H70
1HT1
H62
H38
H63
H65
H30
H69
H23
C56
C60
2HT1
4HT1
C39
Log2FC
HCC
CCA
FDR
NR1H2::RXRA
RXRG
RXRB
MYF6
NR4A2
RORA
NR3C2
NR2F1
TGIF1
ESR2
NR2C2
ELK4
SP8
SPDEF
POU3F3
ETV3
POU3F1
ELK1
POU3F2
POU2F1
SP4
ELF4
POU1F1
POU5F1B
ELF1
KLF14
ELF3
KLF13
ELF5
EHF
Norm. Exprs.
C39 H30 H63 H77 HCC
C56 H38 H65 1HT1 iCCA
C60 H58 H69 2HT1
H23 H62 H70 4HT1
N = 31 TFs
iCCA Adj. Liver
iCCA
HCC Adj. Liver
HCC
p < 2.2x10-6
NR3C2
ELF3
ESR2
SPDEF
ELK1
POU2F1
SP4
NR2F1
RXRA
POU1F1
POU3F2
NR2C2
ELF1
Norm. ES
Sensitivity
1-Specificity
Fold

Supplemental Figure 8 – HCC and iCCA enriched transcription factor motifs

A. UMAP visualization of all malignant cells using gene activity as features for dimensionality reduction and clustering. Bar graph of Log2 fold change of TF motif activity of 31 differentially enriched TF motifs between HCC and iCCA tumors. Colors of bars reflect FDR.

B. Bar graph of Log2 fold change of TF motif activity of 31 differentially enriched TF motifs between HCC and iCCA tumors. Colors of bars reflect FDR.

C. Hierarchical clustering based on malignant cells TF motif activity of top variably active transcription factor motifs.

D. Hierarchical clustering of TIGER-LC cohort gene expression of 31 TFs. Samples are labeled with tumor subtype or adjacent liver.

E. Hierarchical clustering of target genes ES of 13 TFs in the TIGER-LC cohort.

F. ROC curve of TF motif signature for prediction of tumor subtype across each fold in the 5-fold cross-validation training model.

Related to Figure 3 and 4

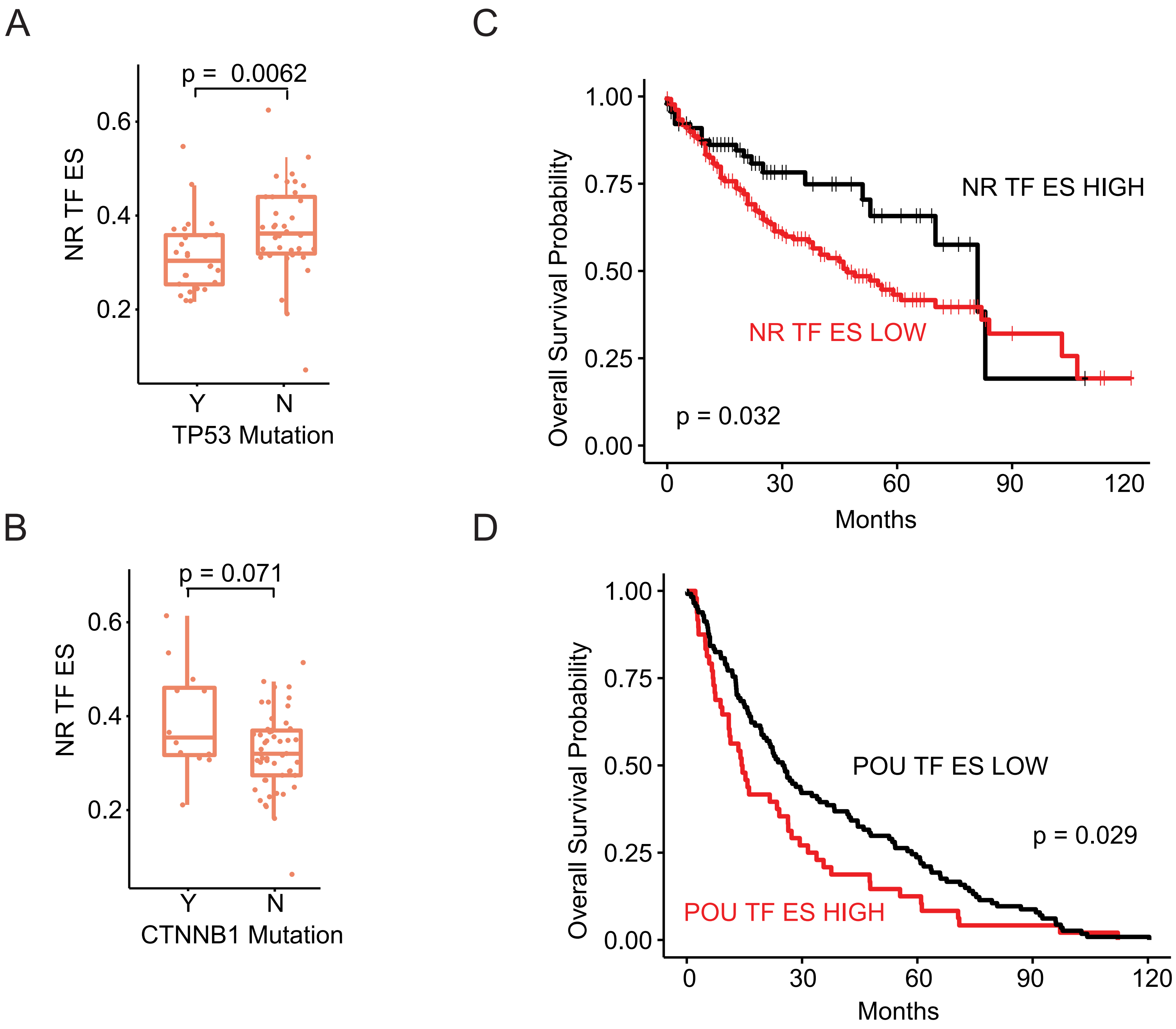
A
p = 0.0062
NR TF ES
0.6
0.4
0.2
Y
N
TP53 Mutation
B
p = 0.071
NR TF ES
0.6
0.4
0.2
Y
N
CTNNB1 Mutation
C
Overall Survival Probability
1.00
0.75
0.50
0.25
0.00
NR TF ES HIGH
NR TF ES LOW
p = 0.032
0
30
60
90
120
Months
D
Overall Survival Probability
1.00
0.75
0.50
0.25
0.00
POU TF ES LOW
p = 0.029
POU TF ES HIGH
0
30
60
90
120
Months

Supplemental Figure 9 – Clinical associations with TF motif enrichment signatures

A. Boxplots of NR ES scores of TIGER-LC cohort of patients with HCC by different TP53 mutation status.

B. Boxplots of NR ES scores of TIGER-LC cohort of patients with HCC by different CTNNB1 mutation status.

C. Kaplan-Meier curves showing the percentage of survival between NR ES high and NR ES low TCGA-LIHC cohort of patients with HCC.

D. Kaplan-Meier curves showing the percentage of survival between POU ES high and POU ES low ICGC cohort of patients with iCCA.

For each boxplot, the center line represents the median. Upper and lower limits of each box represent the 75th and 25th percentiles, respectively.

Related to Figure 6

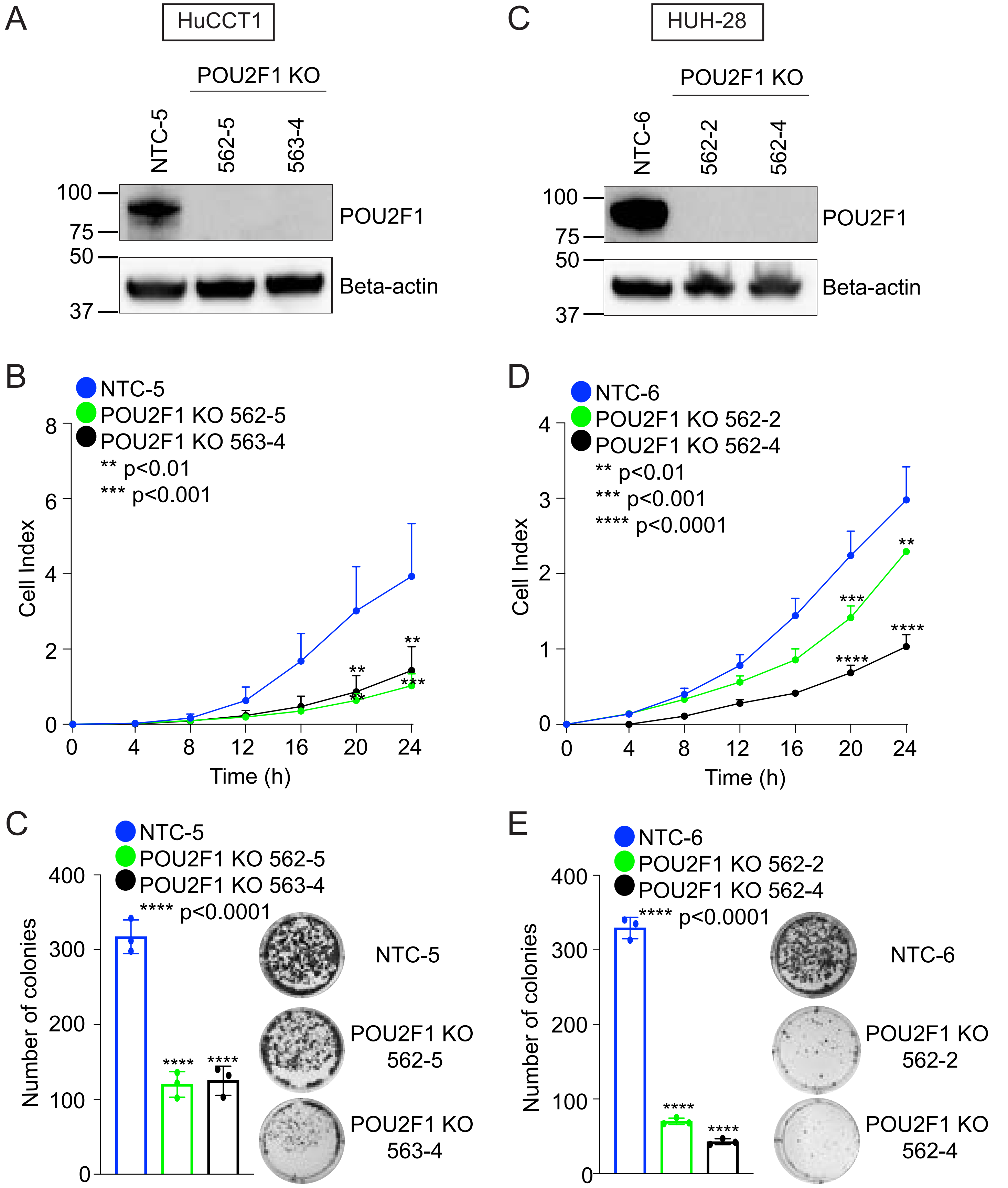
A
HuCCT1
POU2F1 KO
NTC-5
562-5
563-4
100
75
50
37
POU2F1
Beta-actin
C
HUH-28
POU2F1 KO
NTC-6
562-2
562-4
100
75
50
37
POU2F1
Beta-actin
B
NTC-5
POU2F1 KO 562-5
POU2F1 KO 563-4
** p<0.01
*** p<0.001
Cell Index
Time (h)
D
NTC-6
POU2F1 KO 562-2
POU2F1 KO 562-4
** p<0.01
*** p<0.001
**** p<0.0001
Cell Index
Time (h)
C
NTC-5
POU2F1 KO 562-5
POU2F1 KO 563-4
**** p<0.0001
Number of colonies
NTC-5
POU2F1 KO 562-5
POU2F1 KO 563-4
E
NTC-6
POU2F1 KO 562-2
POU2F1 KO 562-4
**** p<0.0001
Number of colonies
NTC-6
POU2F1 KO 562-2
POU2F1 KO 562-4

Supplemental Figure 10 – Functional valitation in-vitro experiments of POU2F1-KO iCCA cell lines

A. Representative immunoblot of HuCCT1 cells with stable POU2F1-KO single cell-derived clones (562-5 and 563-4) and control (NTC-5) of three independent experiments. Corresponding molecular weight markers of each blot were labeled at the left edge of the image.

B. The in vitro cell migration curves of POU2F1-KO single cell-derived clones (562-5 and 563-4) and control (NTC-5) in HuCCT1 cells. Change of impedance is displayed as Cell Index. Mean ± standard error of the mean was calculated from three independent experiments.

C. Clonogenic assay of the effect of POU2F1-KO single cell-derived clones (562-5 and 563-4) and control (NTC-5) in HuCCT1 cells after 10 days. Left - Histogram shows the number of colonies, displayed as Mean ± standard deviation of the mean in three independent experiments. Right - Representative well of three independent experiments.

D. Representative immunoblot of HUH-28 cells with stable POU2F1-KO single cell-derived clones (562-2 and 562-4) and control (NTC-6) of three independent experiments. Corresponding molecular weight markers of each blot were labeled at the left edge of image.

E. The in vitro cell migration curves of POU2F1-KO single cell-derived clones (562-2 and 562-4) and control (NTC-6) in HUH-28 cells. Change of impedance is displayed as Cell Index. Mean ± standard error of the mean was calculated from three independent experiments.

F. Clonogenic assay of the effect of POU2F1-KO single cell-derived clones (562-2 and 562-4) and control (NTC-6) in HUH-28 cells after 10 days. Left - Histogram shows the number of colonies, displayed as Mean ± standard deviation of the mean in three independent experiments. Right - Representative well of three independent experiments.

Statistical significance is determined by 2-way ANOVA test for the migration assay and one-way ANOVA for the clonogenic assay of colony formations considering **$p<0.01$, ***$p<0.001$, ****$p<0.00001$.
